# Vasomotor responses are similar between outbred UM-HET3 and inbred C57BL/6J male and female mouse mesenteric resistance arteries

**DOI:** 10.3389/fphys.2025.1692618

**Published:** 2025-12-01

**Authors:** Robin C. Looft-Wilson, Surya Naraynan, Kathleen Salmon, Ethan Wunibald, Brian Simmons, Connor Haitz, Vishakha Shah, Margaret Xu

**Affiliations:** Department of Health Sciences, William & Mary, Williamsburg, VA, United States

**Keywords:** murine, strain, sex, vasodilation, vasoconstriction, myoendothelial, phenylephrine, eNOS

## Abstract

**Objective:**

Genetically diverse UM-HET3 (HET3) mice have emerged as a more robust model of human large artery dysfunction than the commonly used inbred C57BL/6J (C57) mice. However, HET3 resistance artery function has not been examined. The purpose of this study was to examine HET3 versus C57 mesenteric resistance artery agonist-induced vasomotor responses to phenylephrine (PE) and acetylcholine (ACh), PE-induced myoendothelial feedback (endothelium-dependent feedback dilation to PE-induced vasoconstriction) and its underlying mechanisms, and eNOS (an enzyme involved in endothelium-dependent dilation) expression.

**Hypothesis:**

Vasomotor responses, mechanisms, and eNOS protein expression would be similar between HET3 and C57 mesenteric resistance arteries of both sexes.

**Methods:**

First- and second-order mesenteric arteries from male and female (8–18 weeks old) HET3 and C57 mice were isolated and cannulated for pressure myography. The luminal diameter was measured (in a group-blinded manner) during cumulative addition of PE [10^−9^–10^−5^ M] and then ACh [10^−10^–10^−4^ M]. In separate arteries, myoendothelial feedback was measured by diameter responses (constriction followed by endothelium-dependent feedback dilation) to 10^−5^ M PE over 20 min, ± nitric oxide synthase (NOS) inhibition (10^−5^ M L-NAME) and ± hyperpolarization inhibition with 35 mM KCl to assess myoendothelial feedback mechanisms. eNOS protein expression was measured by Western blot.

**Results:**

Arteries from all groups were similar in size (group mean range: 213–218 µm) and exhibited negligible basal tone (group mean range: 1%–4% constriction). PE-induced peak vasoconstriction (range: 71.0%–73.8% constriction; n = 11–12) and EC-50’s (range: 1.03–1.54 µM) were similar between groups. ACh-induced peak vasodilation (range: 63.1%–73.4% dilation) was also similar between groups. However, ACh EC-50 was significantly (p < 0.05; ANOVA, Bonferroni) lower in HET3 female (0.047 ± 0.021 µM) than in C57 female arteries (4.22 ± 1.97 µM) (p < 0.05). Myoendothelial feedback responses were similar between groups (group mean range: 23.3%–34.0% dilation) at 10 min, but responses were significantly (p < 0.01) greater in HET3 males (56.5% ± 4.9%) than in C57 male arteries (38.8% ± 2.2%) at 20 min (n = 12–15), and they were predominantly dependent on hyperpolarization mechanisms. eNOS/GAPDH and eNOS/total protein expression were similar between the groups.

**Significance:**

In this study, HET3 mesenteric resistance arteries were found to exhibit vasomotor responses similar to those of C57 arteries, with some indications of greater endothelium-dependent vasodilation in HET3, making it a viable mouse model for vascular studies.

## Introduction

The inbred, genetically identical strain C57BL/6 is the most used mouse model in vascular physiology studies (with over 36,000 references on PubMed with the keywords “C57BL” and “vascular”). It has been a useful model of human vascular function; however, there are some differences. For example, C57BL/6 mice do not consistently exhibit hypertension and endothelial dysfunction in response to high salt intake (Sabouri et al., 2024) or diet-induced obesity (DeMarco et al., 2015; Gupte et al., 2008; Police et al., 2009; Symons et al., 2009). This contrasts with humans, who exhibit decreased blood pressure in response to a lower-salt diet or weight loss in overweight individuals (He et al., 2013; Yang et al., 2023).

It has been argued that outbred genetically diverse mice are more appropriate for biomedical research because inbred mice can introduce genetic bias and idiosyncrasies (Miller et al., 1999), and they do not exhibit greater phenotypic stability (Tuttle et al., 2018). For this reason, the UM-HET3 mouse strain (called HET3 hereafter), an outbred, genetically diverse mouse strain (https://www.jax.org/strain/036603), has become a prominent model of human aging. It was developed by breeding CByB6F1/J and C3D2F1/J hybrids together and is used by the National Institute on Aging (NIA) Interventions Testing Program (https://www.nia.nih.gov/research/dab/interventions-testing-program-itp) (Palliyaguru et al., 2021). These mice have been shown to better model the blood pressure changes observed in humans with high-salt (Zheng et al., 2023a) and Western diet-induced obesity (Zheng et al., 2023b). In particular, these studies show that high-salt and Western diets induce increased blood pressure and decreased carotid artery endothelium-dependent dilation in these mice, which is consistent with the effects of high salt and overweight/obesity in humans (Appel, 2017; He et al., 2013; Julius et al., 2000; Kajikawa and Higashi, 2022; Yang et al., 2023).

Although blood pressure control and carotid artery function have been examined in HET3 mice (Zheng et al., 2023a; Zheng et al., 2023b), there has been no examination of resistance artery function in this model. Resistance arteries (generally <500 µm in diameter) are pivotal in control of peripheral vascular resistance (Mulvany and Aalkjaer, 1990) and utilize proportionally different mechanisms for endothelium-dependent vasodilation than large conduit arteries (Jiang et al., 2016; Shimokawa and Godo, 2016; Jiang et al., 2016). For example, conduit arteries rely primarily on nitric oxide for endothelium-dependent vasodilation, whereas resistance arteries rely primarily on endothelium-derived hyperpolarization mechanisms (Shimokawa et al., 1996). Therefore, specific information about resistance artery vasomotor responses is important for validating this strain as a model for vascular physiology studies.

The purpose of this study was to characterize the vasomotor responses of small mesenteric arteries, a resistance artery, in HET3 mice compared to those in the commonly used C57BL/6 mice. We hypothesized that small mesenteric arteries from HET3 mice would have similar vasoconstriction and vasodilation responses as those from C57BL/6 mice. The rationale for this hypothesis is that carotid artery responses to phenylephrine and acetylcholine are similar in these strains (Holly et al., 2023; Zheng et al., 2023a; Zheng et al., 2023b).

We also hypothesized that myoendothelial feedback responses would be similar. Myoendothelial feedback is endothelium-dependent dilation that follows constriction with sympathetic stimulation. It is dependent on α_1_-adrenergic receptor-mediated smooth muscle to endothelial cell gap junction communication and has similar endothelial mechanisms (Dora et al., 1997; Hong et al., 2018; Kerr et al., 2015; Lamboley et al., 2005; Nausch et al., 2012; Tran et al., 2012), namely, nitric oxide and hyperpolarization, as acetylcholine-induced vasodilation (Ding et al., 2000; Waldron et al., 1999). We and others have previously found robust myoendothelial feedback in male C57Bl/6J mouse resistance arteries of various orders, which was dependent on a combination of nitric oxide synthase and hyperpolarization mechanisms (Hong et al., 2018; Looft-Wilson et al., 2017; Looft-Wilson et al., 2024; Nausch et al., 2012). We also recently found that male and female C57Bl/6J mesenteric artery myoendothelial feedback responses and mechanisms are similar between the sexes, with slightly more reliance on nitric oxide mechanisms in male arteries (Looft-Wilson et al., 2024). Because there were minimal sex differences within this strain, we hypothesize that there would also be no strain differences in the myoendothelial feedback response.

In the present study, we found that dose–response relationships to vasomotor agonists and myoendothelial feedback responses to phenylephrine (an α_1_-adrenergic receptor agonist) were similar between the strains and sexes, with similar myoendothelial feedback mechanisms. There were some indications of slightly more robust endothelial responses in HET3 arteries.

## Materials and methods

### Animals

The animal protocol was approved by the William & Mary Institutional Animal Care and Use Committee and is consistent with the Guide for the Care and Use of Laboratory Animals, eighth edition (National Academy of Sciences). A total of 87 mice were used in this study (65 mice in artery function experiments and 22 mice for Western blot assays). The 65 mice used in artery function experiments included 15 male (age range: 56–99 days) and 17 female (age range: 56–120 days) UM-HET3 mice (The Jackson Laboratory, Bar Harbor, MN; strain #036603; RRID:IMSR_JAX:036603), along with 19 male (age range: 59–107 days) and 14 female (age range: 63–126 days) C57Bl/6J mice (The Jackson Laboratory, Bar Harbor, MN; strain #000664; RRID: IMSR_JAX:000664) (Supplementary Table S1 shows ages in each treatment group.). The 22 mice used in Western blot assays included four male and four female (age range: 63–65 days) UM-HET3 mice, along with seven male and seven female (age range: 63–79 days) C57Bl/6J mice. Three of the male and female C57 mice were used for the pooled control sample. Mice were caged by sex (1–3 mice per cage) under a 12:12 h light–dark cycle, with free access to water and standard chow (Teklad LM-485 Mouse/Rat Sterilizable Diet, #T.7012.15; Envigo BioProducts, Inc., Indianapolis, IN), bedding material (Shredded Aspen bedding, #7093, Envigo/Inotiv), and environmental enrichment items (tube or igloo, shredding material, and peanuts in shells).

### Artery dissection and cannulation for vessel function experiments

Mice were euthanized by immersion in a CO_2_ chamber using a gradual displacement of air in the cage with 100% CO_2_ (at 30%–70% displacement/min), and the CO_2_ flow was maintained for >1 min after respiratory arrest. After euthanasia, the intestines and mesenteric bed were removed and placed in a Petri dish of ice-cold MOPS-buffered physiological salt solution (PSS; in mM: 145 NaCl, 4.7 KCl, 2.0 CaCl_2_, 1.17 MgSO_4_, 1.2 NaH_2_PO_4_, 2.0 MOPS, 0.02 EDTA, 5.0 glucose, and 2.0 pyruvate; pH 7.4). One or more first- and/or second-order mesenteric arteries were isolated from each mouse. A total of 233 arteries were used in the vessel function experiments. Most of the perivascular adipose tissue was removed. Dissected arteries were kept in cold PSS until cannulation and generally cannulated within 2 h. Each artery was cannulated using glass micropipettes extending into a stainless-steel organ chamber (Danish Myo Technology A/S, Inc., Aarhus, Denmark) filled with cold PSS and secured to each cannula using 8-0 nylon sutures. Arteries were then pressurized to their approximate *in vivo* pressure [75 mmHg; to approximate *in vivo* pressure and promote myogenic tone (Christensen and Mulvany, 1993; Gros et al., 2002)] by perfusing the lumen with filtered PSS containing 1% BSA [to maintain the glycocalyx (Adamson and Clough, 1992)], with no further luminal flow. Micropipette tips were ∼2/3 the diameter of the arteries when pressurized. The superfusion PSS buffer was not gassed with high oxygen because these arteries have sufficiently thin walls (<15 μm) for efficient oxygen diffusion from the ambient air. Arteries were not aerated with high oxygen to avoid reactive oxygen species generation that can alter endothelium-dependent signaling mechanisms (Wong et al., 2015). The organ chamber was mounted into a culture myograph (model 202 EvB, Danish Myo Technology A/S, Inc.) on a temperature-controlled microscope platform (set to 37 °C). The luminal diameter was measured onscreen from digital images collected with a ×10 objective and an integrated camera, using automatic tracking or manual tracking when automatic tracking was lost (which can happen in very constricted arteries). First-order arteries were used if they were long enough to cannulate, but second-order arteries were more often used because they tended to be longer. After cannulation, arteries were equilibrated for 30 min in the organ chamber with non-recirculated PSS superfusion. Separate arteries were used for agonist dose–response experiments, myoendothelial feedback, and myoendothelial feedback with antagonist experiments. The research worker performing the vessel function experiment was blinded to the strain of the mouse and, in most cases, the sex of the mouse, from which the vessel was isolated.

### Artery dissection for Western blot analysis

Mesenteric arteries (all first- and second-order arteries with fragments of third order) were isolated from individual mice, cleared of most perivascular adipose tissue, frozen in liquid nitrogen, and stored at −80 °C. Arteries were homogenized for Western blot analysis within 21 days of freezing.

### Agonist dose–response

After 30 min of equilibration, the artery was treated with cumulative doses of phenylephrine (PE; 10^−9^–10^−5^ M) and recirculated in a 100 mL reservoir, for 3 min at each dose, to induce progressive constriction. The diameter was measured at 3 min (or peak response) after each dose. After the last dose of PE, diameter changes to cumulative doses of acetylcholine (ACh; 10^−9^–10^−5^ M) were measured at 3 min (or peak response) after each dose.

After PE and ACh measurements, arteries were superfused with Ca^++^-free PSS + EGTA (1 mM) for at least 10 min to determine the maximal diameter in these experiments and all other vessel experiments. In some experiments, the diameter with this treatment was slightly smaller than that at the end of equilibration, so the larger diameter was used as the maximal diameter in calculations.

### Myoendothelial feedback response

After 30 min of equilibration, the artery was treated with phenylephrine (10^−5^ M) recirculated in a 100 mL reservoir to induce constriction. The diameter was recorded every min for 20 min. PE causes a deep constriction in the first 5 min, followed by myoendothelial feedback, which is progressive dilation. This dose of PE induces a large measurable myoendothelial feedback response (Looft-Wilson et al., 2013). PE stimulates alpha_1_-adrenergic receptors, which are the receptors in mouse mesenteric arteries that mediate smooth muscle sympathetic responses and myoendothelial feedback (Nausch et al., 2012; Wei et al., 2018). PE responses are consistent and similar to those induced by the sympathetic neurotransmitter norepinephrine (Perez-Rivera et al., 2004). Acetylcholine (10^−4^ M) was then added to the superfusate, and the artery diameter was measured every min for 15 min to assess agonist-induced endothelium-dependent vasodilation and endothelium viability. The maximal diameter was then measured as described in the previous section.

### NOS and hyperpolarization inhibition during myoendothelial feedback

To test the role of NOS in myoendothelial feedback, arteries were treated with L-NAME (10^−4^ M, or in some cases 10^−3^ M), which was added to the lumen perfusate during cannulation and to the superfusate at the beginning of equilibration, and remained in both buffers throughout the experiment. To test the role of both NOS and hyperpolarization in myoendothelial feedback, separate arteries were treated with 10^−4^ L-NAME and superfused with 35 mM KCl (PSS with equimolar substitution of NaCl) containing 10^−4^ M L-NAME starting at 20 min of equilibration. Myoendothelial feedback measurement was performed as described in the previous section.

### Artery viability criteria

Arteries that did not constrict by at least 50% to PE were considered damaged and were not studied further. Of the 50 arteries cannulated for the dose–response experiments, two experiments were terminated due to little or no constriction to PE, one due to heater failure, and one due to pressure loss. Of the 183 arteries cannulated for myoendothelial feedback experiments, 4 experiments were terminated due to poor/no constriction to PE and 10 experiments due to technical problems (heater malfunction, pressure loss, camera tracking malfunction, or wrong drug added). In three experiments, responses were measured for PE (and included), but acetylcholine responses were lost due to technical issues. Eight vessels that were treated with high L-NAME (10^−3^ M) in the presence or absence of 35 mM KCl were not included in the data due to low numbers in the groups and not being essential to the study.

### Western blotting

Mesenteric artery samples (pooled arteries from one mouse each) were homogenized in 60 µL of lysis buffer with phosphatase inhibitors [50 mM Tris–HCl, 100 mM NaF, 15 mM Na4P2O7, 1 mM Na3VO4, 1% Triton X-100, and 1:200 protease inhibitor cocktail solution (#P2714, Sigma, St. Louis, MO); pH = 7.6], incubated for 1 h at 4 °C, and centrifuged (13,400 rpm, 10 min) to remove insoluble material. Proteins were separated using 10% SDS-PAGE (4% stacking gel) and then electro-transferred to a nitrocellulose membrane. Total protein was visualized on the membrane with ponceau-S to ensure effective transfer. The amount of protein loaded on the SDS-PAGE gels was ∼40 µg/sample, which was estimated based on measuring protein concentration using the Lowry method (DC Protein Assay, Bio-Rad, Hercules, CA) in only a subset of samples to preserve limited protein. Membranes were then blocked for 1 h at room temperature using a LiCor Intercept Blocking Buffer (#927-70001, LiCor, Lincoln, NE) and incubated overnight with an eNOS antibody (BD Transduction Laboratories, Franklin Lakes, NJ, #610297, mouse monoclonal, 1:1,000) diluted in LiCor Intercept Antibody Diluent (#927-75001), followed by four washes of 5 min each with PBS +0.1% Tween-20. Subsequently, the membranes were incubated for 45 min with a near-infrared secondary antibody [goat anti-mouse IRDye 800 CW (#926-32210), 1:20,000, LiCor] diluted in LiCor Intercept Antibody Diluent, then washed again four times for 5 min each with PBS +0.1% Tween-20, and finally rinsed and stored in PBS. Bands were visualized, and density was measured using the LiCor Odyssey System. Membranes were then labeled with a GAPDH antibody (Millipore, #MAB374, mouse monoclonal, 1:1,000) using the same protocol as for eNOS labeling. Finally, the membrane was stained for total protein using the Revert 700 Total Protein Stain (LiCor, #926–11011), which was visualized and quantified using the LiCor Odyssey System. Protein sizes are as follows: ∼140 KDa for eNOS and ∼36 KDa for GAPDH. Both antibodies labeled a prominent band of the predicted molecular weight, and we previously showed that these antibodies and the protein stain have a linear signal in the range of 25–50 µg of mouse mesenteric artery protein (Looft-Wilson et al., 2024). The specificity of the eNOS antibody was demonstrated in our previous paper by the near elimination of the eNOS signal following denudation [Figure 5; Looft-Wilson et al. (2013)].

### Data analysis

The maximal diameter was determined using Ca^++^-free PSS, unless a greater diameter was observed pre- or post-equilibration, in which case the greater value was used. Basal tone was determined as the fraction of the diameter at post-equilibration relative to the maximal diameter. Basal tone was also calculated at 20 min of equilibration (“Pre-KCl”) for the group treated with 10^−4^ M L-NAME +35 mM KCl to allow comparison of basal tone before and after KCl addition (“Post-KCl”). EC-50 doses were calculated with a nonlinear 3-factor curve fit using GraphPad Prism software (San Diego, CA). Responses to PE at each time-point were calculated as follows: % constriction = (diameter at each time-point − post-equilibration diameter)/post-equilibration diameter * 100. The “post-equilibration diameter” is the diameter at the end of the initial 30-min equilibration and immediately before the addition of PE. Occasionally, a min measurement was lost during the time-course measurements due to tracking or camera issues, and in this case (22 instances across all experiments and time-points), this value was interpolated. Percent constriction responses to PE in the group treated with 10^−4^ M L-NAME +35 mM KCl were calculated using the diameter at 20 min equilibration (before addition of KCl) as the post-equilibration diameter to show the relative magnitude of baseline constriction with each inhibitor. Responses to ACh at each time-point were calculated as follows: % dilation = [diameter at each time-point−pre-ACh diameter]/[maximal diameter–pre-ACh diameter] * 100. Responses to PE and ACh for the control treatment were also calculated as follows: % of maximal diameter = diameter at each time-point/maximal diameter * 100. Myoendothelial feedback responses to PE at 10 and 20 min were calculated as follows: % dilation = (diameter at 10 min or 20 min − diameter at peak constriction)/(maximal diameter − diameter at peak constriction) * 100. Statistical analysis was performed using GraphPad Prism software. Maximal diameter, basal tone, peak constriction, peak dilation, and myoendothelial feedback at 10 min and 20 min were compared between each strain and sex and between treatments within each strain/sex group using one-way ANOVA, along with the Bonferroni *post hoc* test if the group effect was significant. Time-course responses to phenylephrine and acetylcholine were compared separately between the strain/sex groups and between each treatment within a strain/sex group using a repeated-measures two-way ANOVA with Geisser–Greenhouse correction, and the Bonferroni *post hoc* analysis of each time-point was performed if the group effect was significant. The exception was the time-course responses expressed as a % of the maximal diameter, which included both phenylephrine and acetylcholine in a single graph and were analyzed using a mixed-effects model with Geisser–Greenhouse correction and Tukey’s *post hoc* test (due to missing data for acetylcholine response in one subject in each of two groups).

Western blot band intensity values were determined using LiCor Odyssey software by drawing a box around the band and subtracting the mean background signal directly above and below the band (Supplementary Figure S4). The intensity value for eNOS was normalized to the GAPDH band intensity value and the total protein lane intensity value. These ratios (eNOS/ GAPDH and eNOS/total protein) were then normalized to the ratio of the same control sample (pooled mesenteric artery protein from three C57 male and three C57 female individuals) on each blot to allow comparison between blots and combination of the blots (Figure 8). The normalized expression of each gene was compared between the groups using a one-way ANOVA.

## Results

**FIGURE 1:**
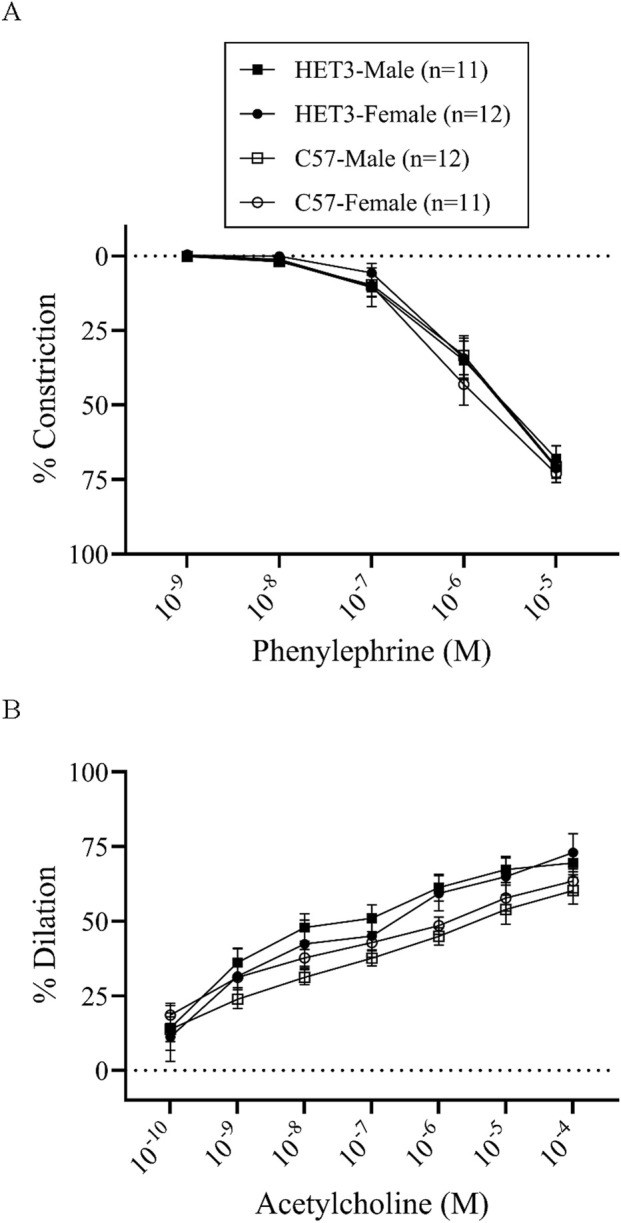
Cumulative dose–response curves (% constriction relative to baseline diameter; means ± SEM) to phenylephrine superfusion (10^−9^–10^−5^ M; 3 min per dose) **(A)**, followed by cumulative dose–response curves (% dilation, relative to pre-constricted and maximal diameters, means ± SE) to acetylcholine superfusion (10^−10^–10^−4^ M; 3 min per dose) **(B)**, were not different between male and female HET3 and C57 mouse mesenteric arteries. Responses were compared using two-way repeated-measures ANOVA (nonsignificant).

In the dose–response experiments, artery sizes, basal tone, peak constriction to PE, and peak dilation to ACh were similar between all groups (Table 1). Cumulative dose–response curves to PE and ACh were similar between all groups (Figure 1). However, the sensitivity to ACh was significantly greater (P < 0.05) in arteries from HET3 female mice than in those from C57 female mice, as indicated by a lower EC-50 value in HET3 female mice (Table 1).

**TABLE 1:** Artery dimensions and dose–responses to phenylephrine (PE) (10^−9^–10^−5^ M) and acetylcholine (ACh) (10^−10^–10^−4^ M) (mean ± SEM).

	HET3 male	HET3 female	C57 male	C57 female
	(n = 11)	(n = 12)	(n = 12)	(n = 11)
Maximal luminal diameter (µm)	210 ± 7	216 ± 6	217 ± 8	212 ± 12
Basal tone (fraction of maximal diameter)	0.99 ± 0.00	0.98 ± 0.01	0.97 ± 0.01	0.97 ± 0.02
EC-50 for PE (M)	1.33*10^−6^ ± 3.49*10^−7^	1.50*10^−6^ ± 3.71*10^−7^	1.54*10^−6^ ± 3.42*10^−7^	1.03*10^−6^ ± 2.85*10^−7^
EC-50 for ACh (M)	8.08*10^−7^ ± 7.73*10^−7^	4.70*10^−8^ ± 2.08*10^−8^	4.30*10^−7^ ± 1.91*10^−7^	4.22*10^−6^ ± 1.97*10^−6^†
Peak constriction to phenylephrine (%)	70.8 ± 3.7	71.2 ± 3.4	72.5 ± 3.0	73.6 ± 2.9
Peak dilation to acetylcholine (%)	72.2 ± 4.6	73.0 ± 6.3	63.1 ± 5.2	68.2 ± 4.5

Group values were compared using one-way ANOVA, with the Bonferroni *post hoc* test.

^†^p < 0.05 compared to HET3 female.

EC-50 values were calculated using nonlinear (3-factor) regression curve fitting.

**FIGURE 2:**
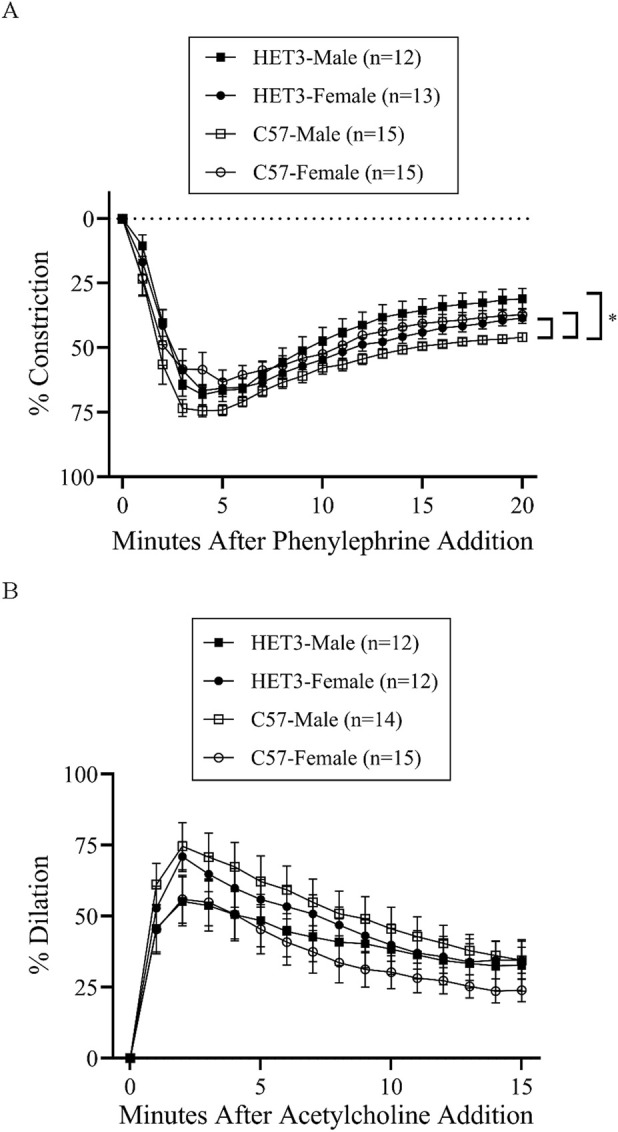
Responses (% constriction relative to the baseline diameter; means ± SEM) to a single dose of phenylephrine superfusion (10^−5^ M) over time **(A)** resulted in a deep constriction followed by an attenuated constriction (myoendothelial feedback), which was significantly different in C57 male arteries compared to other groups (*P < 0.05; significant time-points and P-values are listed in Supplementary Table S2A). Subsequent responses (% dilation, relative to pre-constricted and maximal diameters, means ± SE) to acetylcholine superfusion (10^−4^ M), in the presence of continued phenylephrine superfusion (10^−5^ M), over time **(B)** were not significantly different between the groups. Group responses were compared using two-way repeated-measures ANOVA with the Bonferroni *post hoc* test.

In the myoendothelial feedback experiments, all groups in the control treatment had similar maximal diameters and little or no basal tone (Table 2). Peak constriction to PE and peak dilation to ACh were also similar between these groups (Table 3). The time-dependent response to PE was significantly different between HET3 male and C57 male arteries, with less constriction in HET3 male arteries at 16–20 min (Figure 2A; Supplementary Table S2A), which corresponded to a greater calculated myoendothelial feedback response in HET3 male at 20 min (Figure 7B; Table 4), but not at 10 min (Figure 7A; Table 4). C57 male arteries also exhibited significantly greater constriction than C57 female arteries at 15 and 18–20 min, and greater constriction than HET3 female arteries at 3 min (Figure 2A; Supplementary Table S2A). However, there were no differences in myoendothelial feedback at 10 or 20 min between these groups (Figure 7; Table 4). The dynamic response to ACh was not different between these groups (Figure 2B).

**TABLE 2:** Dimensions (mean ± SEM) of arteries used in myoendothelial feedback experiments.

	HET3 male	HET3 female	C57 male	C57 female
Maximal luminal diameter (µm)				
Control	230 ± 9 (n = 12)	206 ± 9 (n = 13)	217 ± 9 (n = 15)	209 ± 7 (n = 15)
L-NAME (10^−4^ M)	211 ± 5 (n = 13)	215 ± 6 (n = 16)	212 ± 9 (n = 9)	227 ± 12 (n = 12)
L-NAME (10^−4^ M) + 35 mM KCl	192 ± 11 (n = 7)††	212 ± 10 (n = 11)	222 ± 12 (n = 8)	244 ± 13 (n = 6)
Basal tone (fraction of maximal diameter)				
Control	0.99 ± 0.00 (n = 12)	0.99 ± 0.00 (n = 13)	0.98 ± 0.01 (n = 15)	0.98 ± 0.01 (n = 15)
L-NAME (10^−4^ M)	0.99 ± 0.00 (n = 13)	0.99 ± 0.00 (n = 16)	0.94 ± 0.02 (n = 9)	0.95 ± 0.02 (n = 12)
L-NAME (10^−4^ M) + 35 mM KCl (3.5 × 10^−2^) (Pre-KCl)	0.98 ± 01 (n = 7)	0.99 ± 0.00 (n = 11)	0.98 ± 0.01 (n = 8)	0.97 ± 0.02 (n = 6)
L-NAME (10^−4^ M) + 35 mM KCl (3.5 × 10^−2^) (Post-KCl)	0.64 ± 0.06 (n = 7)††††	0.68 ± 0.05 (n = 11)††††	0.64 ± 0.08 (n = 8)††††	0.56 ± 0.06 (n = 6)††††

Control values between groups were compared using one-way ANOVA, with the Bonferroni *post hoc* test.

No significant differences were found.

L-NAME treatments were compared to control within each group using one-way ANOVA with the Bonferroni *post hoc* test.

^††^p < 0.01 and ^††††^p < 0.0001 compared to control within the respective group.

**TABLE 3:** Peak vasomotor responses (mean ± SEM) of arteries used in myoendothelial feedback experiments.

	HET3 male	HET3 female	C57 male	C57 female
Peak constriction to 10^−5^ M PE (% of basal diameter)				
Control	72.0 ± 3.3 (n = 12)	71.2 ± 1.4 (n = 13)	76.8 ± 1.9 (n = 15)	74.6 ± 2.4 (n = 15)
L-NAME (10^−4^ M)	78.2 ± 1.8 (n = 13)	81.0 ± 1.0 (n = 16)††††	75.8 ± 1.9 (n = 9)	74.1 ± 3.3 (n = 12)
L-NAME (10^−4^ M) + 35 mM KCl	83.9 ± 1.5 (n = 7)†	82.7 ± 1.9 (n = 11)††††	82.4 ± 3.1 (n = 8)	82.3 ± 2.0 (n = 6)
Peak dilation to 10^−4^ M ACh (%)				
Control	64.5 ± 8.7 (n = 12)	76.4 ± 4.0 (n = 13)	78.8 ± 7.5 (n = 14)	64.2 ± 7.4 (n = 15)
L-NAME (10^−4^ M)	46.7 ± 7.5 (n = 13)	71.3 ± 5.6 (n = 16)	65.3 ± 5.8 (n = 9)	75.1 ± 9.3 (n = 12)
L-NAME (10^−4^ M) + 35 mM KCl	19.4 ± 4.6 (n = 7)††	21.1 ± 2.9 (n = 11)††††	18.9 ± 5.6 (n = 8)††††	22.2 ± 6.7 (n = 6)††

Control values between groups were compared using one-way ANOVA, with the Bonferroni *post hoc* test.

No significant differences were found.

L-NAME treatments were compared to control within each group using one-way ANOVA, with the Bonferroni *post hoc* test.

^†^p < 0.05, ^††^p < 0.01, and ^††††^p < 0.0001 compared to control within the respective group.

**TABLE 4:** Myoendothelial feedback responses (mean ± SEM).

	HET3 male	HET3 female	C57 male	C57 female
Myoendothelial feedback at 10 min (% dilation)				
Control	34.0 ± 5.7 (n = 12)	23.3 ± 2.9 (n = 13)	24.0 ± 3.1 (n = 15)	28.1 ± 4.8 (n = 15)
L-NAME (10^−4^ M)	26.7 ± 5.9 (n = 13)	30.6 ± 7.0 (n = 16)	18.0 ± 4.9 (n = 9)	12.9 ± 4.2 (n = 12)†
L-NAME (10^−4^ M) + 35 mM KCl	14.8 ± 5.4 (n = 7)	17.2 ± 4.5 (n = 11)	7.7 ± 3.0 (n = 8)††	6.0 ± 2.3 (n = 6)†
Myoendothelial feedback at 20 min (% dilation)				
Control	56.5 ± 4.9 (n = 12)	45.3 ± 3.0 (n = 13)	38.8 ± 2.2 (n = 15)**	47.8 ± 3.9 (n = 15)
L-NAME (10^−4^ M)	56.6 ± 4.8 (n = 13)	57.8 ± 6.5 (n = 16)	37.0 ± 6.9 (n = 9)	40.7 ± 3.9 (n = 12)
L-NAME (10^−4^ M) + 35 mM KCl	21.1 ± 4.3 (n = 7)†††	29.3 ± 5.6 (n = 11)	11.0 ± 6.1 (n = 8) †††	9.0 ± 4.1 (n = 6)††††

Control values between groups were compared using one-way ANOVA, with the Bonferroni *post hoc* test.

**p < 0.01 compared to HET3 male control.

L-NAME treatments were compared to control within each group using one-way ANOVA, with the Bonferroni *post hoc* test.

^†^p < 0.05, ^††^p < 0.01, ^†††^p < 0.001, and ^††††^p < 0.0001 compared to control within the respective group.

**FIGURE 3:**
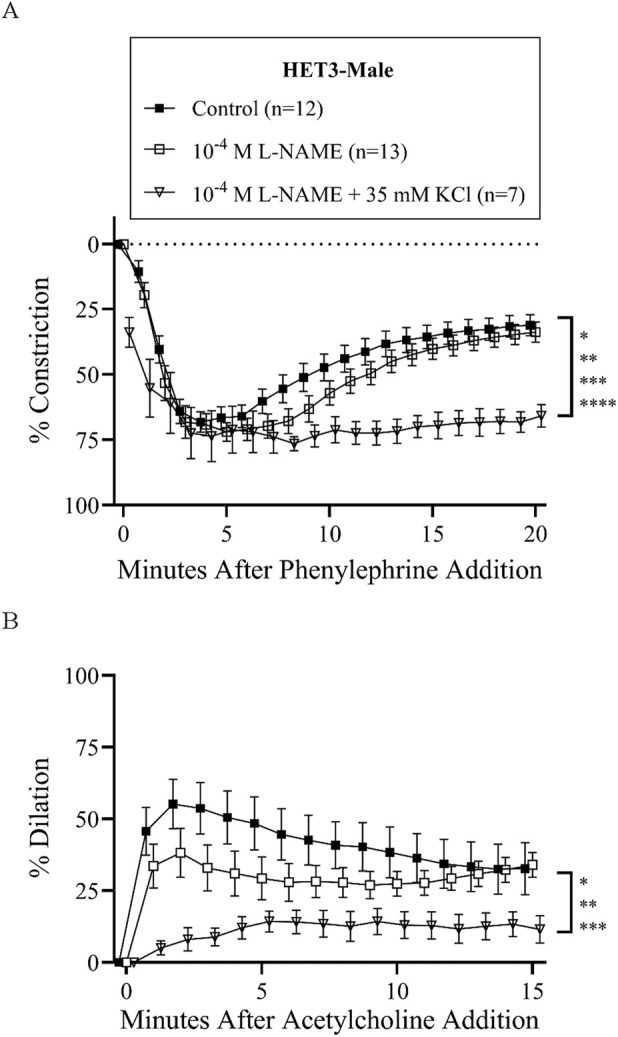
HET3 male mesenteric resistance artery responses (% constriction relative to baseline diameter; means ± SEM) to a single dose of phenylephrine superfusion (10^−5^ M) **(A)** indicate that the addition of antagonists of nitric oxide synthases (NOS) and hyperpolarization mechanisms (10^−4^ M L-NAME +35 mM KCl) significantly inhibit myoendothelial feedback, but NOS inhibition alone (10^−4^ M L-NAME) does not (*P < 0.05, **P < 0.01, ***P < 0.001, and ****P < 0.0001; significant time-points and P-values are listed in Supplementary Table S2B). Subsequent responses (% dilation, relative to pre-constricted and maximal diameters, means ± SE) to acetylcholine superfusion (10^−4^ M), in the presence of continued phenylephrine superfusion (10^−5^ M), over time **(B)** were also only significantly inhibited by both NOS and hyperpolarization inhibition (*P < 0.05, **P < 0.01, and ***P < 0.001; significant time-points and P-values are listed in Supplementary Table S2C). Group responses were compared using two-way repeated-measures ANOVA with the Bonferroni *post hoc* test. “Control” group data are the same as “HET3-male” group data in Figure 2.

**FIGURE 4:**
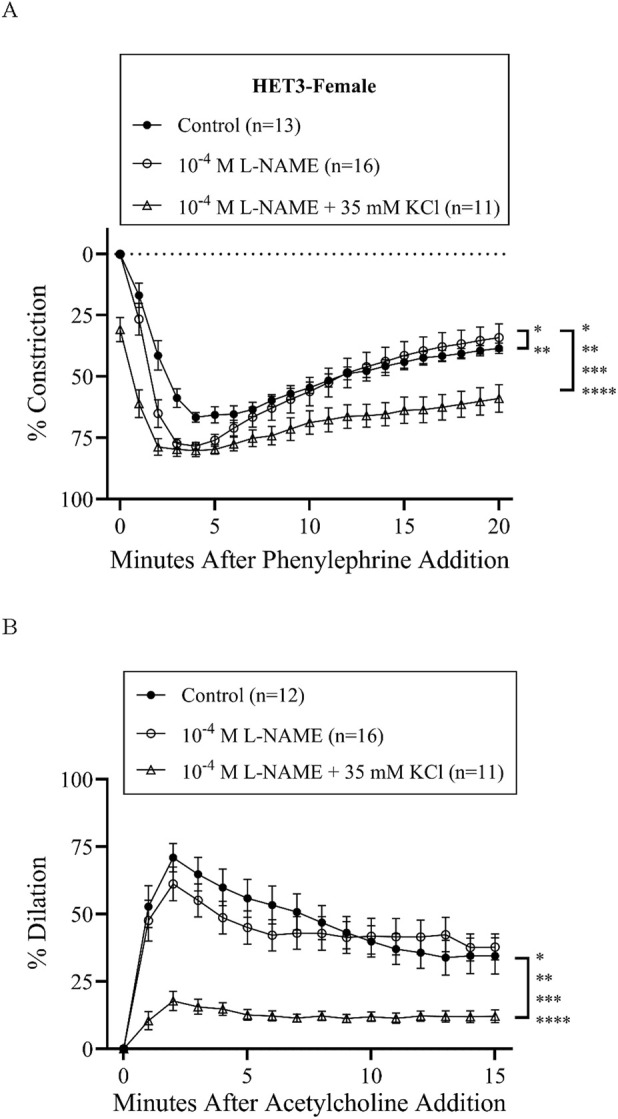
HET3 female mesenteric resistance responses (% constriction relative to the baseline diameter; means ± SEM) to a single dose of phenylephrine superfusion (10^−5^ M) **(A)** indicate that the addition of antagonists to nitric oxide synthases (NOSs) (10^−4^ M L-NAME) significantly enhances initial constriction, and NOS + hyperpolarization antagonists (10^−4^ M L-NAME +35 mM KCl) significantly inhibit myoendothelial feedback (*P < 0.05, **P < 0.01, ***P < 0.001, and ****P < 0.0001; significant time-points and P-values are listed in Supplementary Table S2D). Subsequent responses (% dilation, relative to pre-constricted and maximal diameters, means ± SE) to acetylcholine superfusion (10^−4^ M), in the presence of continued phenylephrine superfusion (10^−5^ M), over time **(B)** were also only significantly inhibited by both NOS and hyperpolarization inhibition (*P < 0.05, **P < 0.01, ***P < 0.001, and ****P < 0.0001; significant time-points and P-values are listed in Supplementary Table S2E). Group responses were compared using two-way repeated-measures ANOVA with Bonferroni *post hoc* test. Acetylcholine response for one artery in the “Control” group is missing due to technical issues. “Control” group data are the same as “HET3-female” group data in Figure 2.

**FIGURE 5:**
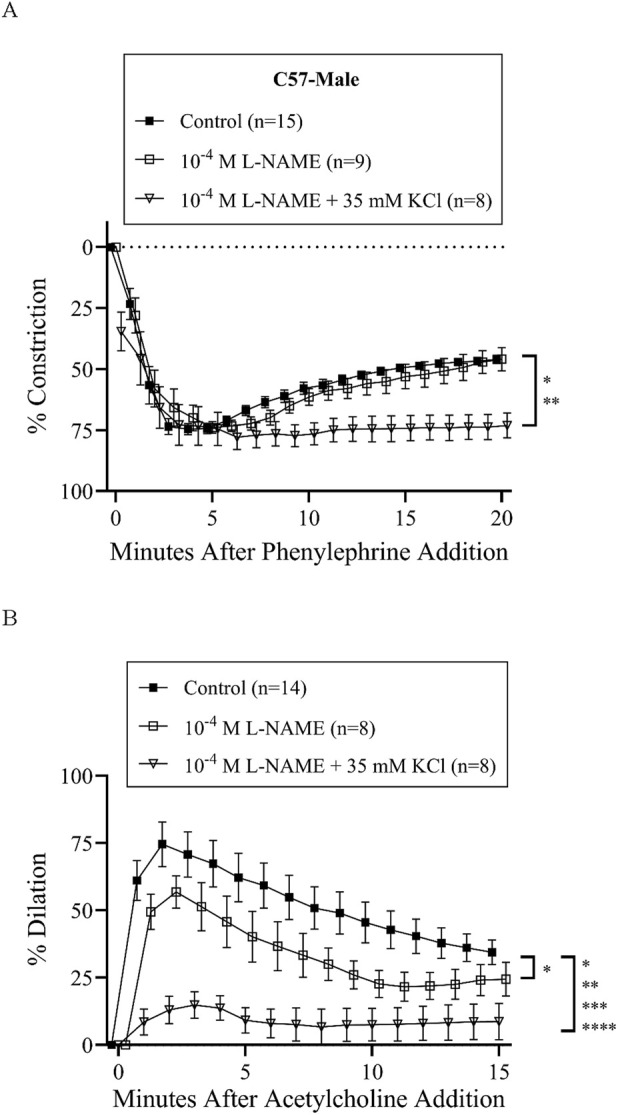
C57 male mesenteric resistance artery responses (% constriction relative to the baseline diameter; means ± SEM) to a single dose of phenylephrine superfusion (10^−5^ M) **(A)** indicate that the addition of antagonists of nitric oxide synthases (NOSs) and hyperpolarization mechanisms (10^−4^ M L-NAME +35 mM KCl) significantly inhibit myoendothelial feedback, but NOS inhibition alone (10^−4^ M L-NAME) does not (*P < 0.05 and **P < 0.01; significant time-points and P-values are listed in Supplementary Table S2F). Subsequent responses (% dilation, relative to pre-constricted and maximal diameters, means ± SE) to acetylcholine superfusion (10^−4^ M), in the presence of continued phenylephrine superfusion (10^−5^ M), over time **(B)** were significantly inhibited by both inhibitor treatment groups (*P < 0.05, **P < 0.01, ***P < 0.001, and ***P < 0.001; significant time-points and P-values are listed in Supplementary Table S2G). Group responses were compared using two-way repeated-measures ANOVA with Bonferroni *post hoc* test. Acetylcholine response for one artery in the “Control” group and one artery in the 10^−4^ L-NAME group are missing due to technical issues. “Control” group data are the same as “C57-male” group data in Figure 2.

**FIGURE 6:**
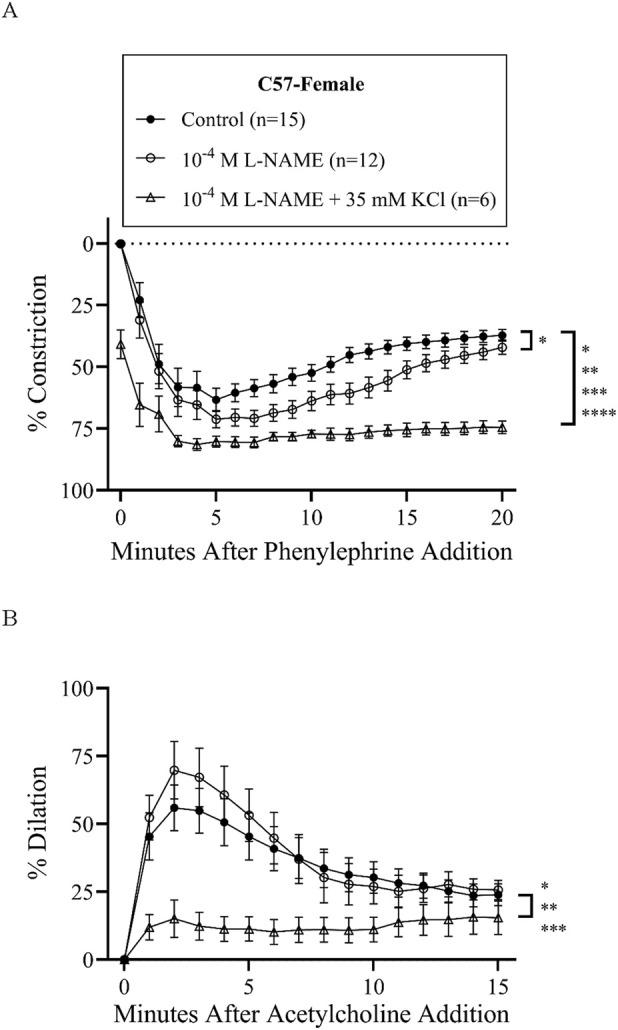
C57 female mesenteric resistance artery responses (% constriction relative to baseline diameter; means ± SEM) to a single dose of phenylephrine superfusion (10^−5^ M) **(A)** indicate that the addition of antagonists of nitric oxide synthases (NOSs) (10^−4^ M L-NAME) and NOS + hyperpolarization mechanisms (10^−4^ M L-NAME +35 mM KCl) significantly inhibit myoendothelial feedback (*P < 0.05, **P < 0.01, ***P < 0.001, and ***P < 0.001; significant time-points and P-values are listed in Supplementary Table S2H). Subsequent responses (% dilation, relative to pre-constricted and maximal diameters, means ± SE) to acetylcholine superfusion (10^−4^ M), in the presence of continued phenylephrine superfusion (10^−5^ M), over time **(B)** were significantly inhibited only by NOS + hyperpolarization inhibition (*P < 0.05, **P < 0.01, ***P < 0.001, and ***P < 0.001; significant time-points and P-values are listed in Supplementary Table S2I). Group responses were compared using two-way repeated-measures ANOVA with Bonferroni *post hoc* test. “Control” group data are the same as “C57-female” group data in Figure 2.

**FIGURE 7:**
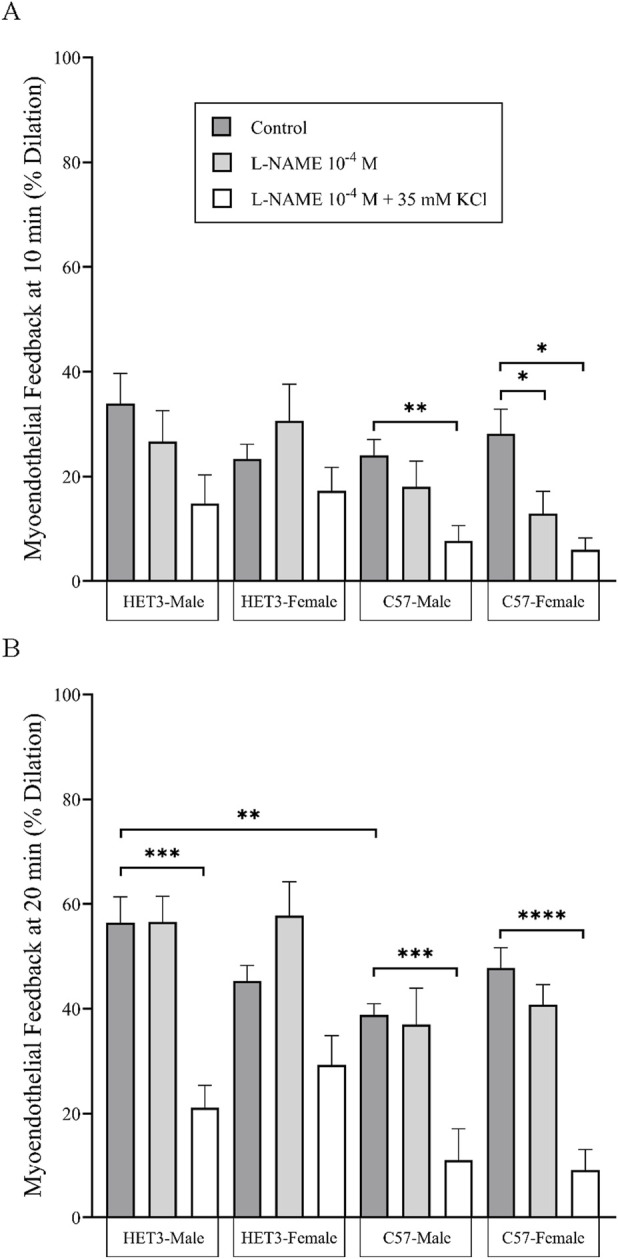
Myoendothelial feedback responses (% dilation relative to diameter at peak constriction and maximal diameter; means ± SEM) of mesenteric resistance arteries in control conditions were not different between strains and sexes at 10 min **(A)** but were significantly greater (**P < 0.01) in HET3 male than in C57 male arteries at 20 min **(B)**. Treatment with nitric oxide synthase antagonist (10^−4^ M L-NAME) only significantly inhibited myoendothelial feedback in C57 female arteries at 10 min **(A)**, and treatment with NOS + hyperpolarization antagonists (10^−4^ M L-NAME +35 mM KCl) significantly inhibited myoendothelial feedback in C57 arteries at 10 min **(A)** and all groups, except for HET3 female arteries at 20 min **(B)** (*P < 0.05, **P < 0.01, ***P < 0.001, and ***P < 0.001). Groups and treatments within each group were each compared using one-way ANOVA with the Bonferroni *post hoc* test. The values in this figure are also displayed in Table 4.

Treatment with L-NAME (10^−4^ M) to block nitric oxide synthases, most importantly eNOS, did not affect basal tone in any groups (Table 2). There was virtually no tone with this treatment. This treatment results in slightly more constriction at many time-points with phenylephrine treatment in all groups (Figures 3A, 4A, 5A, 6A), but constriction was only significantly different from the control treatment in the HET3 female and C57 female groups (Figures 4A, 6A; Supplementary Tables S2D, S2H). Peak constriction to phenylephrine was also significantly greater with L-NAME than the control treatment in the HET3 female group, but not in other groups (Table 3). Myoendothelial feedback was significantly decreased by L-NAME in the C57 female group compared to the control treatment at 10 min, but not at 20 min, and not in any other groups (Table 4; Figure 7). Treatment with L-NAME did not affect peak vasodilation to acetylcholine compared to control treatment in any group (Table 3) and only decreased the time-dependent response to acetylcholine in C57 male arteries at 9–10 min (Figure 5; Supplementary Table S2G).

Treatment with L-NAME (10^−4^ M) and 35 mM KCl to block both nitric oxide and hyperpolarization mechanisms resulted in significant basal tone in all groups, which can be attributed to the KCl treatment because it was added after incubation with L-NAME, which did not enhance tone (Table 2). This treatment significantly increased peak constriction to phenylephrine compared to control treatment in HET3 arteries, but not in C57 arteries (Table 3). The time-dependent constriction to phenylephrine was significantly enhanced in all groups at most time-points (Figures 3A, 4A, 5A, 6A; Supplementary Tables S2B, D, F, H). Myoendothelial feedback was significantly decreased compared to control treatment at 10 min in C57 male and C57 female groups and at 20 min in all groups, except for the HET3 female group (Table 4; Figure 7). Treatment with L-NAME and KCl nearly eliminated dilation to acetylcholine at almost all time-points in all groups compared to control treatment (Figures 3B, 4B, 5B, 6B; Supplementary Tables S2C, E, G, I) and significantly decreased peak dilation to acetylcholine in all groups (Table 3).

**FIGURE 8:**
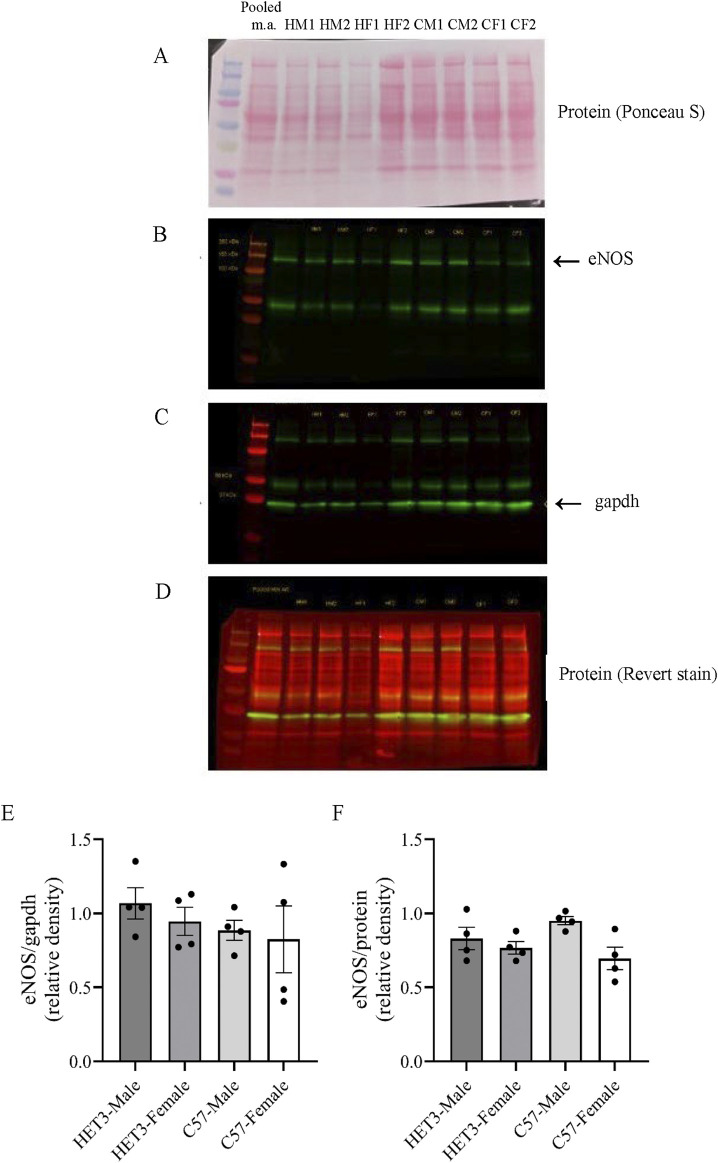
Western blot analysis of eNOS protein expression normalized to GAPDH and total protein indicate similar expression across male and female HET3 and C57 mouse mesenteric arteries (n = 4 in each group). One representative membrane (with two samples per group and a pooled mesenteric artery control consistent across membranes) illustrates that membranes were labeled for ponceau S protein stain **(A)**, followed by labeling with an eNOS antibody **(B)**, then a GAPDH antibody **(C)**, and then LiCor Revert protein stain **(D)**. Quantification of bands for all membranes are shown in Supplementary Figure S4. Group values of eNOS/ GAPDH **(E)** and eNOS/protein **(F)** were normalized to the values of the pooled mesenteric artery sample on each blot and compared using one-way ANOVA (no significant difference).

Protein expression of eNOS in whole first- and second-order mesenteric arteries was similar between groups whether normalized to GAPDH or total protein (Figure 8).

## Discussion

This study found that mesenteric resistance arteries from HET3 mice exhibited vasomotor responses similar to those from C57 mice. However, HET3 artery responses were slightly greater in two aspects. Sensitivity to ACh was significantly greater in female HET3 arteries than in female C57 arteries, and myoendothelial feedback responses were significantly greater in male HET3 than in male C57 arteries at 20 min. This suggests that vasodilatory responses in HET3 mesenteric resistance arteries are slightly more robust than those of C57 mice. These results add to the evidence that the HET3 mouse model is valid and possibly slightly superior to the standard C57 mouse model for vascular studies (Zheng et al., 2023b; Zheng et al., 2023a), given that greater endothelial cell function generally indicates better vascular health (Gutierrez et al., 2013; Vanhoutte et al., 2017). Different inbred strains of mice have been shown to exhibit varying vasomotor responses, with C57Bl/6J mice having a greater response to the endothelium-dependent vasodilator acetylcholine (an indication of robust endothelial function) than most inbred strains (Holly et al., 2023; Kim et al., 2016). Inbred strains, however, are more susceptible to genetic and phenotypic idiosyncrasies (Miller et al., 1999) and do not completely mimic the vascular pathology of humans. Therefore, the validation of normal HET3 resistance artery function, together with the more human-like vascular pathology (Zheng et al., 2023a; Zheng et al., 2023b), indicates that this outbred strain may be an improvement over inbred strains in the translational potential of vascular studies.

This was the first study to examine myoendothelial feedback responses in a mouse strain other than C57Bl/6J (Hong et al., 2018; Looft-Wilson et al., 2017; Looft-Wilson et al., 2024; Nausch et al., 2012). Myoendothelial feedback has been examined in isolated arteries from hamster, rat, pig, and mouse (Lemmey et al., 2020), along with *in vivo* in humans, in which it is described as “sympathetic escape” (Hearon and Dinenno, 2019). It seems to play an important role in blood pressure regulation in animals (Battault et al., 2018; Cao et al., 2020) and tissue blood flow in humans (Barcroft et al., 1954; Joyner et al., 1990). Moreover, mesenteric arteries (as used in this study) exhibit sympathetic escape *in vivo* (Folkow et al., 1964). In this study, we verify that myoendothelial feedback is present and is robust in mesenteric arteries of the HET3 strain, and of similar magnitude to the C57Bl/6J strain. Thus, the response is not an artifact of the C57Bl/6J strain.

The mechanism of myoendothelial feedback in various arteries has been reported to involve the generation of inositol triphosphate (IP_3_) and increased intracellular Ca^++^ in smooth muscle during alpha_1_-adrenergic receptor activation (Lemmey et al., 2020). One or both of these molecules diffuse to the endothelial cells through gap junctions to activate eNOS either directly or indirectly through IP_3_-induced release of endoplasmic reticulum Ca^++^ and cause nitric oxide production, which relaxes smooth muscle (Lemmey et al., 2020). The increased endothelial Ca^++^ also activates Ca^++^-activated K^+^ channels (IK_Ca_ and SK_Ca_), which causes endothelial hyperpolarization and subsequent smooth muscle hyperpolarization and relaxation (Lemmey et al., 2020). The relative importance of nitric oxide and hyperpolarization varies between artery types (Lemmey et al., 2020). In the present study, both myoendothelial feedback to phenylephrine and endothelium-dependent vasodilation to ACh were found to be mostly due to hyperpolarization in all groups, with very little contribution from nitric oxide. This was the most surprising finding in this study because previous studies of male C57Bl/6J first- and second-order mesenteric arteries from our laboratory have shown a more prominent contribution from nitric oxide in myoendothelial feedback (Looft-Wilson et al., 2013; Looft-Wilson et al., 2024). Two other laboratories examined a smaller branch order (third) of these arteries and found that hyperpolarization was the predominant myoendothelial feedback mechanism (Hong et al., 2018; Nausch et al., 2012). So, in the present study, these first- and second-order arteries responded more like the third-order arteries in other studies. It is known that endothelium-dependent mechanisms in the mesenteric artery network become more dependent upon hyperpolarization as the branch order becomes smaller and myoendothelial gap junctions become more abundant (Shimokawa et al., 1996). Moreover, the overall endothelium-dependent mechanisms have been recently shown to vary between first- and second-order mouse mesenteric arteries (Zhang et al., 2025). Based on the branching pattern and size, we presume to be studying predominantly second-order mesenteric arteries. However, the mesenteric vascular bed exhibits variable branching patterns among individual mice, making it difficult to standardize. Therefore, we cannot be certain that the precise structure/order is consistent across vessels/subjects. It is also possible that the strain and/or the experimental conditions, although presumed to be consistent with our previous studies, have some drift over time. We considered the possibility that the dose of L-NAME (10^−4^ M) was not sufficient, so we also tested a greater dose (10^−3^ M) in male and female C57Bl/6J mice (Supplementary Figures S1, S2). The phenylephrine responses to both doses of L-NAME were similar, so it is unlikely that the lack of response to L-NAME was due to insufficient NOS antagonism.

The inhibition of both nitric oxide synthases and hyperpolarization (with L-NAME +35 mM KCl) eliminated most, but not all, of the myoendothelial feedback response. Residual myoendothelial feedback was most prominent in the female HET3 arteries (Figure 4A; Table 4). Some possible mechanisms for this are other endothelium-dependent mechanisms (e.g., prostacyclin release) or smooth muscle-dependent factors, such as desensitization of the smooth muscle to phenylephrine. However, we believe that endothelium-dependent factors are the most likely because in a previous paper, we found that endothelium denudation eliminated endothelial feedback (Looft-Wilson et al., 2013). We did not replicate the denudation experiments because we have recently found it difficult to denude the endothelium without killing the artery completely even when using several different techniques.

### Limitations

The dose–response experiments were performed by measuring response to each dose at the end of 3 min. This protocol has some limitations. Not all responses were necessarily stable at 3 min, so it could underestimate the response to some doses. Moreover, the responses to the lower doses of ACh could be continued myoendothelial feedback to the last dose of PE, rather than a response to the very low initial doses of ACh. It is not possible to distinguish between these two possibilities. However, because the first doses of ACh occurred within 10 min of the greatest dose of PE, we suspect that the response to at least the lowest dose or two of ACh is primarily, if not entirely, due to myoendothelial feedback. This can affect the interpretation of the acetylcholine dose–response curves and EC-50 values because it is not clear how much of this response is due to ACh.

Although it appears that the acetylcholine responses are weak when expressed as % dilation (Figures 1B, 2B), this can be misleading because a significant amount of dilation occurred prior to the addition of acetylcholine due to the myoendothelial feedback response. When expressed as % of the maximal diameter, the time course of responses to phenylephrine, followed by acetylcholine, showed a deep constriction (<50% of maximal diameter at peak constriction in all arteries), followed by peak dilation generally greater than 80% of the maximal diameter (Supplementary Figure S3; Table 5). This indicates the expected amount of endothelium-dependent vasodilation normally expected in a healthy resistance artery *in vitro*.

**TABLE 5:** Peak responses to phenylephrine (10^−5^ M) and acetylcholine (10^−4^ M) expressed as % of the maximal diameter.

	HET3 male	HET3 female	C57 male	C57 female
Dose–response experiments				
Peak response to 10^−5^ M phenylephrine	27.9 ± 3.9 (n = 11)	26.9 ± 3.5 (n = 12)	25.0 ± 2.7 (n = 12)	23.3 ± 2.4 (n = 11)
Peak response to 10^−4^ M acetylcholine	80.7 ± 3.2 (n = 11)	81.2 ± 3.7 (n = 12)	73.5 ± 3.9 (n = 12)	75.8 ± 3.7 (n = 11)
Control myoendothelial feedback experiments				
Peak response to 10^−5^ M phenylephrine	26.8 ± 3.4 (n = 12)	28.2 ± 1.5 (n = 13)	20.8 ± 1.6 (n = 15)	23.3 ± 2.5 (n = 15)
Peak response to 10^−4^ M acetylcholine	89.0 ± 3.1 (n = 12)	91.2 ± 1.3 (n = 13)	90.7 ± 3.0 (n = 14)	86.7 ± 2.9 (n = 15)

Groups were compared using one-way ANOVA, with the Bonferroni *post hoc* test.

No significant differences were found.

Responses were calculated as diameter at each time-point/maximal diameter*100.

The combination of first- and second-order arteries we used in this study could potentially introduce variability in vascular responses. So, we compared the responses of the largest and smallest arteries from the group with the greatest range in artery size (C57 male; control group, myoendothelial feedback experiments). These arteries did not significantly differ in phenylephrine or acetylcholine responses (Supplementary Figure S5). Therefore, it is unlikely that this was a significant source of variability.

Although all mice used in this study were within the age of young adult of 28 days–6 months (Barros et al., 2021), some treatment groups had arteries from a wider age range of mice. For example, the HET3 female group in the control myoendothelial feedback treatment was significantly older than the other groups for this treatment although the average age was a trivial 24 days older than the youngest group average. This group also had the greatest range in age between vessels within the group. Therefore, we compared responses to phenylephrine and acetylcholine in the HET3 female arteries from the three youngest mice (age 60–78 days) and the three oldest mice (120 days) in this group (Supplementary Figure S6) and found no difference. Therefore, we do believe that the age range used in this study is a confounding factor.

## Conclusion

This study indicates that vasomotor responses of mesenteric resistance arteries from HET3 mice of both sexes are equal to or slightly greater than those of C57 mice. Vasoconstriction to phenylephrine and vasodilation to the endothelium-dependent vasodilator are similar between groups, with some indication of greater sensitivity to acetylcholine in HET3 female than in C57 female arteries. Myoendothelial feedback, an important endothelium-dependent response, is slightly greater in male HET3 than in male C57 arteries, and the mechanism is predominantly due to hyperpolarization in all groups. Therefore, HET3 mesenteric resistance arteries are a robust model of vascular function that may represent an advantage over the C57 mouse model, given the better overall vascular translational potential of the strain.

## Data Availability

The raw data supporting the conclusions of this article will be made available by the authors, without undue reservation.
