# Supplementary material for: Vasomotor responses are similar between outbred UM-HET3 and inbred C57BL/6J male and female mouse mesenteric resistance arteries
Source: Front Physiol. 2025 Dec 1;16:1692618. doi: 10.3389/fphys.2025.1692618 (PMC12702883; doi:10.3389/fphys.2025.1692618)

Supplementary Table 1. Ages in days (mean ± SEM) of mice in each treatment group. Ages were compared between strain/sex groups by one-way ANOVA with Bon Ferroni post-hoc test for the "Dose-Response" experiment and the "Control" treatment in the "Myoendothelial Feedback" experiment. This same statistical test was also performed across "Myoendothelial Feedback" treatments (compared to "Control" treatment) within each strain/sex group. ** $p<0.01$, *** $p<0.001$ compared to Control HET3-Female. † $p<0.05$, †† $p<0.01$, ††† $p<0.001$, †††† $p<0.0001$ compared to Control within the respective strain/sex group.

Experiment	HET3-Male	HET3-Female	C57- Male	C57-Female
Dose-Response	77 ± 4 (n=12)	71 ± 3 (n=13)	69 ± 1 (n=15)	68 ± 1 (n=15)
Myoendothelial Feedback				
Control	73 ± 4 (n=12)**	94 ± 5 (n=13)	70 ± 3 (n=15)***	70 ± 2 (n=15)***
L-NAME (10^{-4} M)	68 ± 2 (n=13)	71 ± 3 (n=16)†††	83 ± 4 (n=9)	97 ± 6 (n=12)††††
L-NAME (10^{-4} M) + 35 mM KCl	72 ± 3 (n=7)	79 ± 2 (n=11)†	91 ± 7 (n=8)††	115 ± 5 (n=6)††††

Supplementary Table 2. Significance levels (P-values) at each time-point between groups or between treatments within a group that had overall significant differences (Figs. 2A, 3A, 3B, 4A, 4B, 5A, 5B, 6A, 6B). Determined by two-way repeated measures ANOVA with Bonferroni post-hoc tests. Significant differences highlighted in gray.

A

**Control Responses to Phenylephrine Between Groups**	Minutes after Phenylephrine (10^{-5} M) Addition									
**RM-ANOVA Group Diff: P=0.0077 (Figure 2A)**	1	2	3	4	5	6	7	8	9	10
HET3-Male (n=12) vs. HET3-Female (n=13)	>0.9999	>0.9999	>0.9999	>0.9999	>0.9999	>0.9999	>0.9999	>0.9999	>0.9999	>0.9999
HET3-Male (n=12) vs. C57-Male (n=15)	0.6351	0.563	0.6856	>0.9999	0.7761	>0.9999	>0.9999	>0.9999	0.6892	0.516
HET3-Male (n=12) vs. C57-Female (n=15)	0.8616	>0.9999	>0.9999	>0.9999	>0.9999	>0.9999	>0.9999	>0.9999	>0.9999	>0.9999
HET3-Female (n=13) vs. C57-Male (n=15)	>0.9999	0.8013	0.0441	0.0877	0.259	>0.9999	>0.9999	>0.9999	>0.9999	>0.9999
HET3-Female (n=13) vs. C57-Female (n=15)	>0.9999	>0.9999	>0.9999	>0.9999	>0.9999	>0.9999	>0.9999	>0.9999	>0.9999	>0.9999
C57-Male (n=15) vs. C57-Female (n=15)	>0.9999	>0.9999	0.5306	0.2267	0.2882	0.1144	0.3061	0.8433	0.7298	>0.9999

	11	12	13	14	15	16	17	18	19	20
HET3-Male (n=12) vs. HET3-Female (n=13)	>0.9999	>0.9999	0.6466	0.6939	0.695	0.6709	0.5978	0.6299	0.61	0.7233
HET3-Male (n=12) vs. C57-Male (n=15)	0.2497	0.1741	0.1	0.0899	0.0679	0.0374	0.0385	0.036	0.0226	0.0243
HET3-Male (n=12) vs. C57-Female (n=15)	>0.9999	>0.9999	>0.9999	>0.9999	>0.9999	>0.9999	>0.9999	>0.9999	>0.9999	>0.9999
HET3-Female (n=13) vs. C57-Male (n=15)	>0.9999	0.6065	0.9232	0.629	0.4882	0.2155	0.2165	0.111	0.0637	0.068
HET3-Female (n=13) vs. C57-Female (n=15)	>0.9999	>0.9999	>0.9999	>0.9999	>0.9999	>0.9999	>0.9999	>0.9999	>0.9999	>0.9999
C57-Male (n=15) vs. C57-Female (n=15)	0.4274	0.1115	0.1211	0.0636	0.0491	0.0601	0.0796	0.0499	0.03	0.0337

B

**HET3-Male Responses to Phenylephrine with/without Antagonists**	Minutes after Phenylephrine (10^{-5} M) Addition									
**RM-ANOVA Group Diff: P=0.0002 (Figure 3A)**	1	2	3	4	5	6	7	8	9	10
Control (n=12) vs. 10^{-4} M L-NAME (n=13)	0.3384	0.2799	>0.9999	>0.9999	0.6766	0.8361	0.3239	0.188	0.2303	0.3454
Control (n=12) vs. 10^{-4} M L-NAME + 35 mM KCl (n=7)	0.0112	0.2905	0.9236	>0.9999	>0.9999	>0.9999	0.2103	0.0057	0.0085	0.0088

	11	12	13	14	15	16	17	18	19	20
Control (n=12) vs. 10^{-4} M L-NAME (n=13)	0.454	0.47	0.6403	0.771	0.9202	0.8561	>0.9999	>0.9999	>0.9999	>0.9999
Control (n=12) vs. 10^{-4} M L-NAME + 35 mM KCl (n=7)	0.0012	0.0006	0.0003	0.0002	0.0004	0.0002	0.0003	0.0001	<0.0001	<0.0001

C

HET3-Male Responses to Acetylcholine with/without Antagonists RM-ANOVA Group Diff: P=0.0181 (Figure 3B)	Minutes after Acetylcholine (10^{-4} M) Addition									
	1	2	3	4	5	6	7	8	9	10
Control (n=12) vs. 10^{-4} M L-NAME (n=13)	0.585	0.3479	0.1938	0.2414	0.2445	0.2935	0.3569	0.3897	0.3713	0.5619
Control (n=12) vs. 10^{-4} M L-NAME + 35 mM KCl (n=7)	0.0009	0.0003	0.0007	0.0035	0.008	0.0145	0.0188	0.0195	0.0309	0.0444

	11	12	13	14	15
Control (n=12) vs. 10^{-4} M L-NAME (n=13)	0.7553	>0.9999	>0.9999	>0.9999	>0.9999
Control (n=12) vs. 10^{-4} M L-NAME + 35 mM KCl (n=7)	0.0559	0.0699	0.1012	0.1356	0.1114

D

HET3-Female Responses to Phenylephrine with/without Antagonists RM-ANOVA Group Diff: P=0.0009 (Figure 4A)	Minutes after Phenylephrine (10^{-5} M) Addition									
	1	2	3	4	5	6	7	8	9	10
Control (n=13) vs. 10^{-4} M L-NAME (n=16)	0.4879	0.0156	0.0008	0.0002	0.0378	0.4788	>0.9999	>0.9999	>0.9999	>0.9999
Control (n=13) vs. 10^{-4} M L-NAME + 35 mM KCl (n=11)	<0.0001	<0.0001	0.0005	0.0008	0.0034	0.0246	0.0332	0.0106	0.0268	0.0341

	11	12	13	14	15	16	17	18	19	20
Control (n=13) vs. 10^{-4} M L-NAME (n=16)	>0.9999	>0.9999	>0.9999	>0.9999	>0.9999	>0.9999	>0.9999	>0.9999	0.983	0.9192
Control (n=13) vs. 10^{-4} M L-NAME + 35 mM KCl (n=11)	0.0223	0.0111	0.0074	0.0041	0.0095	0.0051	0.0048	0.0061	0.0087	0.0096

E

HET3-Female Responses to Acetylcholine with/without Antagonists RM-ANOVA Group Diff: P=0.0001 (Figure 4B)	Minutes after Acetylcholine (10^{-4} M) Addition									
	1	2	3	4	5	6	7	8	9	10
Control (n=12) vs. 10^{-4} M L-NAME (n=16)	>0.9999	0.487	0.5527	0.4632	0.5149	0.4629	0.7875	>0.9999	>0.9999	>0.9999
Control (n=12) vs. 10^{-4} M L-NAME + 35 mM KCl (n=11)	0.0003	<0.0001	<0.0001	<0.0001	0.0001	0.0002	0.0002	0.0003	0.0004	0.0009

	11	12	13	14	15
Control (n=12) vs. 10^{-4} M L-NAME (n=16)	>0.9999	>0.9999	0.7078	>0.9999	>0.9999
Control (n=12) vs. 10^{-4} M L-NAME + 35 mM KCl (n=11)	0.0016	0.0039	0.0134	0.0124	0.0145

F

**C57-Male Responses to Phenylephrine with/without Antagonists**	Minutes after Phenylephrine (10^{-5} M) Addition									
**RM-ANOVA Group Diff: P=0.0002 (Figure 5A)**	1	2	3	4	5	6	7	8	9	10
Control (n=15) vs. 10^{-4} M L-NAME (n=9)	>0.9999	>0.9999	0.7386	0.8744	>0.9999	0.9614	0.2689	0.2351	0.6625	0.8191
Control (n=15) vs. 10^{-4} M L-NAME + 35 mM KCl (n=8)	0.2041	0.8698	>0.9999	>0.9999	>0.9999	0.4676	0.202	0.0791	0.0174	0.0079

	11	12	13	14	15	16	17	18	19	20
Control (n=15) vs. 10^{-4} M L-NAME (n=9)	>0.9999	0.9426	>0.9999	0.8542	>0.9999	>0.9999	>0.9999	>0.9999	>0.9999	>0.9999
Control (n=15) vs. 10^{-4} M L-NAME + 35 mM KCl (n=8)	0.0128	0.0091	0.0072	0.0049	0.0034	0.0027	0.0024	0.0018	0.0018	0.0017

G

**C57-Male Responses to Acetylcholine with/without Antagonists**	Minutes after Acetylcholine (10^{-4} M) Addition									
**RM-ANOVA Group Diff: P=0.0002 (Figure 5B)**	1	2	3	4	5	6	7	8	9	10
Control (n=14) vs. 10^{-4} M L-NAME (n=8)	0.504	0.1988	0.2625	0.2234	0.2169	0.1715	0.1577	0.0968	0.0472	0.0426
Control (n=14) vs. 10^{-4} M L-NAME + 35 mM KCl (n=8)	<0.0001	<0.0001	<0.0001	<0.0001	0.0001	<0.0001	0.0003	0.0007	0.0009	0.0017

	11	12	13	14	15
Control (n=14) vs. 10^{-4} M L-NAME (n=8)	0.053	0.0673	0.1374	0.2745	0.4292
Control (n=14) vs. 10^{-4} M L-NAME + 35 mM KCl (n=8)	0.0027	0.0041	0.0068	0.0097	0.0142

H

**C57-Female Responses to Phenylephrine with/without Antagonists**	Minutes after Phenylephrine (10^{-5} M) Addition									
**RM-ANOVA Group Diff: P=0.0001 (Figure 6A)**	1	2	3	4	5	6	7	8	9	10
Control (n=15) vs. 10^{-4} M L-NAME (n=12)	0.8646	>0.9999	>0.9999	0.9	0.3733	0.1144	0.0311	0.0519	0.0287	0.0752
Control (n=15) vs. 10^{-4} M L-NAME + 35 mM KCl (n=6)	0.0054	0.1583	0.0313	0.0099	0.0078	0.0003	<0.0001	<0.0001	<0.0001	<0.0001

	11	12	13	14	15	16	17	18	19	20
Control (n=15) vs. 10^{-4} M L-NAME (n=12)	0.0588	0.0143	0.0209	0.0271	0.0573	0.1303	0.1844	0.2096	0.2402	0.4186
Control (n=15) vs. 10^{-4} M L-NAME + 35 mM KCl (n=6)	<0.0001	<0.0001	<0.0001	<0.0001	<0.0001	<0.0001	<0.0001	<0.0001	<0.0001	<0.0001

I

C57-Female Responses to Acetylcholine with/without Antagonists RM-ANOVA Group Diff: P=0.0386 (Figure 6B)	Minutes after Acetylcholine (10^{-4} M) Addition 1	2	3	4	5	6	7	8	9	10
Control (n=15) vs. 10^{-4} M L-NAME (n=12)	>0.9999	0.6316	0.7469	0.9357	>0.9999	>0.9999	>0.9999	>0.9999	>0.9999	>0.9999
Control (n=15) vs. 10^{-4} M L-NAME + 35 mM KCl (n=6)	0.0062	0.003	0.0006	0.0015	0.0049	0.0079	0.0142	0.0297	0.0334	0.0357

	11	12	13	14	15
Control (n=15) vs. 10^{-4} M L-NAME (n=12)	>0.9999	>0.9999	>0.9999	>0.9999	>0.9999
Control (n=15) vs. 10^{-4} M L-NAME + 35 mM KCl (n=6)	0.1438	0.2199	0.3769	0.6404	0.5589

J

Control Responses to Phenylephrine (% of Maximal Diameter) Mixed Effects Model Group Diff: P=0.0287 (Supplementary Figure 3)	Minutes after Phenylephrine (10^{-5} M) Addition 1	2	3	4	5	6	7	8	9	10
HET3-Male (n=12) vs. HET3-Female (n=13)	0.7943	>0.9999	0.7609	0.9652	0.9929	0.9972	0.9618	0.9147	0.792	0.6442
HET3-Male (n=12) vs. C57-Male (n=15)	0.2979	0.2564	0.2678	0.3427	0.2658	0.5495	0.4341	0.408	0.2789	0.2163
HET3-Male (n=12) vs. C57-Female (n=15)	0.3829	0.7598	0.954	0.6967	0.9841	0.8592	0.9996	0.9822	0.9284	0.7686
HET3-Female (n=13) vs. C57-Male (n=15)	0.7563	0.3248	0.0113	0.006	0.0546	0.2519	0.4768	0.4006	0.3684	0.4831
HET3-Female (n=13) vs. C57-Female (n=15)	0.8187	0.8126	0.9994	0.7873	0.999	0.9072	0.8826	0.9887	0.986	0.9988
C57-Male (n=15) vs. C57-Female (n=15)	>0.9999	0.8954	0.3117	0.1466	0.1501	0.0528	0.161	0.4101	0.3883	0.5844

	11	12	13	14	15	16	17	18	19	20
HET3-Male (n=12) vs. HET3-Female (n=13)	0.6129	0.6181	0.4061	0.4309	0.4343	0.4295	0.392	0.4094	0.4039	0.4523
HET3-Male (n=12) vs. C57-Male (n=15)	0.111	0.0784	0.0462	0.0419	0.0316	0.0179	0.0183	0.0171	0.0112	0.0119
HET3-Male (n=12) vs. C57-Female (n=15)	0.7548	0.8347	0.6871	0.6847	0.6686	0.5647	0.5393	0.5642	0.4768	0.4617
HET3-Female (n=13) vs. C57-Male (n=15)	0.2723	0.1438	0.196	0.1206	0.0858	0.0352	0.0318	0.012	0.007	0.0075
HET3-Female (n=13) vs. C57-Female (n=15)	0.9942	0.9589	0.9298	0.9327	0.9493	0.9946	0.9946	0.9943	0.9998	>0.9999
C57-Male (n=15) vs. C57-Female (n=15)	0.2611	0.077	0.08	0.0394	0.0292	0.0374	0.0511	0.0333	0.0201	0.0234

Supplementary Figure 1. C57-Male mesenteric resistance artery responses (% constriction relative to baseline diameter; means ± SE) to a single dose of phenylephrine superfusion (10^{-5} M) with or without addition of two different doses (10^{-4} M or 10^{-3} M) of nitric oxide synthases (NOS) antagonist L-NAME indicate no significant difference in responses between the two doses, and statistically similar responses as Control. Group responses were compared by 2-way repeated-measures ANOVA. "Control" group data is the same as "C57-Male" group data in Figure 2 and "Control" group data in Figure 5. "10^{-4} L-NAME" group data is the same data as shown in Figure 5.

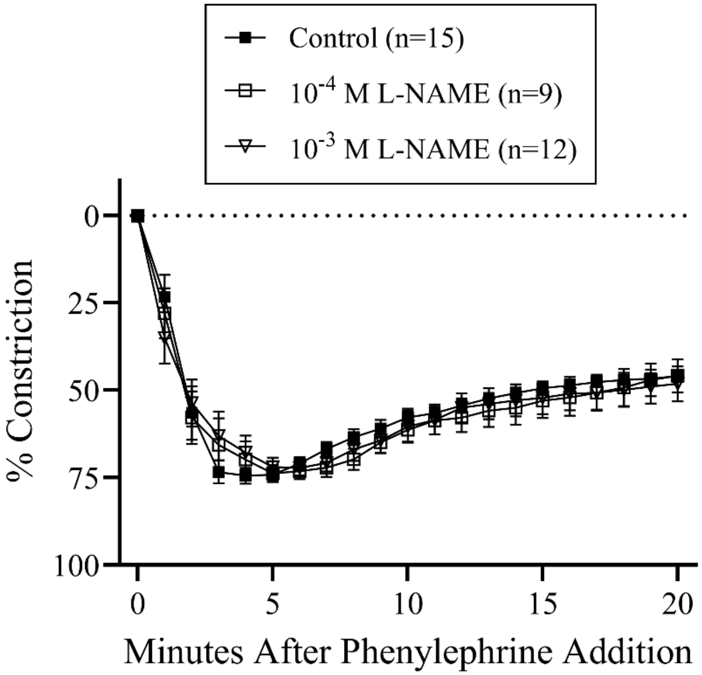

Supplementary Figure 2. C57-Female mesenteric resistance artery responses (% constriction relative to baseline diameter; means ± SE) to a single dose of phenylephrine superfusion (10^{-5} M) with or without addition of two different doses (10^{-4} M or 10^{-3} M) of nitric oxide synthases (NOS) antagonist L-NAME indicate no significant difference in responses between the two doses, but statistically greater constriction (*P<0.05) compared to Control with 10^{-4} M L-NAME at mins 7, 9, 12-14, and with 10^{-3} M L-NAME at mins 9-11. Group responses were compared by 2-way repeated-measures ANOVA with Bon Ferroni post-hoc test. "Control" group data is the same as "C57-Female" group data in Figure 2 and "Control" group data in Figure 6. "10^{-4} L-NAME" group data is the same data as shown in Figure 6.

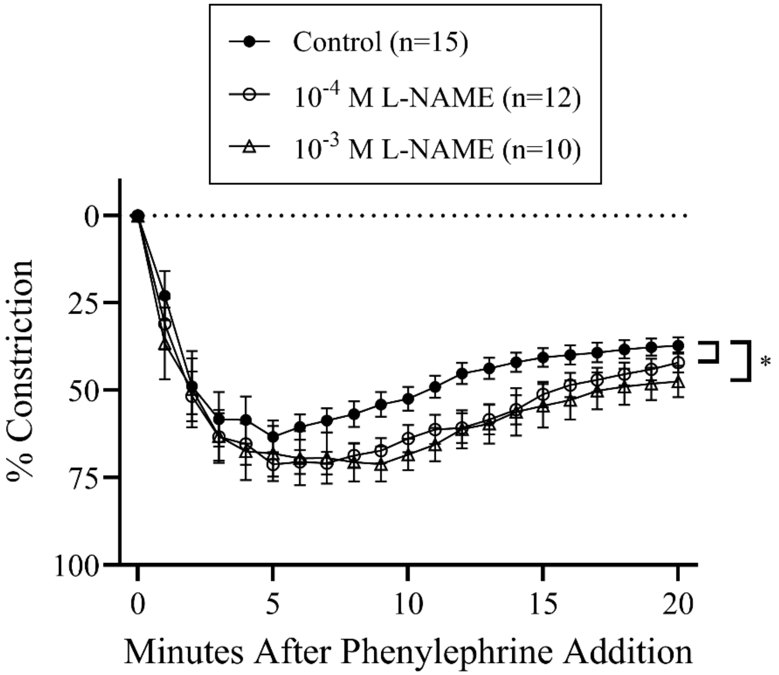

Supplementary Figure 3. Responses (% of maximal diameter; means ± SE) to a single dose of phenylephrine superfusion (10^{-5} M) over time (0-20 min) resulted in deeper constriction C57-Male arteries compared to other groups (*P<0.05, **P<0.01; Significant time-points and P-values are listed in Supplementary Table 1J), but no differences in responses to subsequent acetylcholine superfusion (10^{-4} M) (20-35 min), in the presence of continued phenylephrine superfusion (10^{-5} M). Group responses were compared by Mixed Effects Model with Tukey post-hoc test (due to missing data for acetylcholine response in HET3-Female and C57-Male groups). Number of subjects for HET3-Female and C57-Male groups are listed in the figure legend for the phenylephrine and acetylcholine responses, respectively.

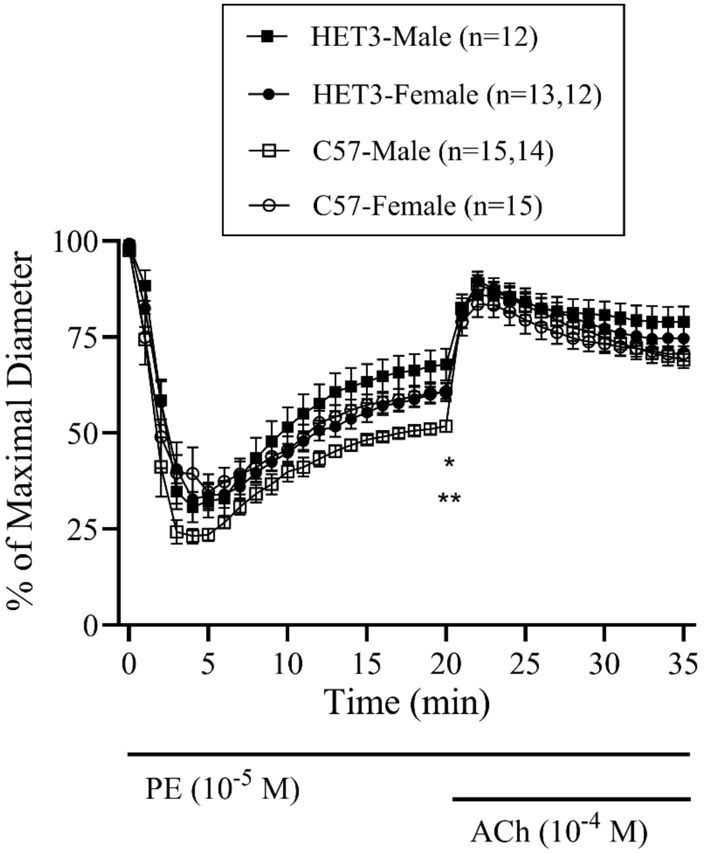

Supplementary Figure 4. Quantification of western blot membranes for eNOS, gapdh, and total protein across groups (male and female HET3 and C57 mice) using near-infra red imaging. There are 3 blots with a total 4 samples per group (2 samples per group per blot and a pooled mesenteric artery sample that serves as a normalizing sample across blots). Blots #1 and Blots #3 contain the same samples in a different order and the values were averaged for analysis in Figure 8 (panels F and G). Each sample lane has ~40 µg of protein. HM = HET3-Male, HF = HET3-Female, CM = C57-Male, CF = C57-Female. Protein quantification was performed using more than one image position (with the same size quantification box in each lane) to optimize the inclusion of the bands in each lane within the quantification box. Background subtraction for each band was calculated using average signal within the space directly above and below each box marked by the pale dotted lines.

Blot #1 (Lanes: ladder, pooled mesenteric artery, HM1, HM2, HF1, HF2, CM1, CM2, CF1, CF2)

eNOS

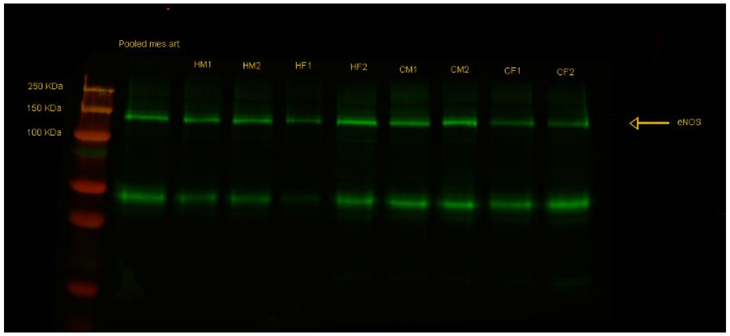

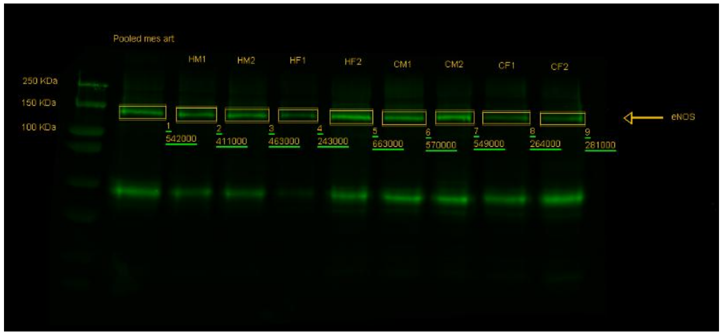

gapdh

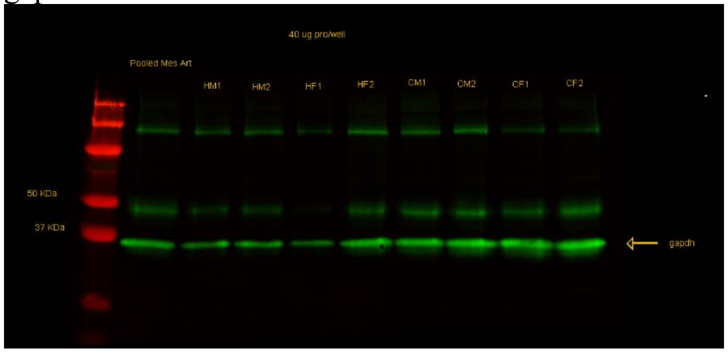

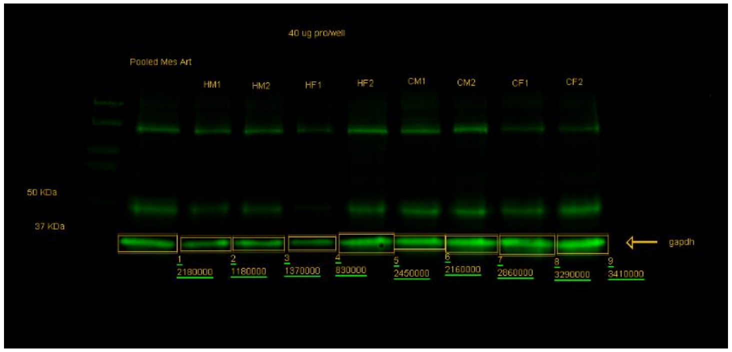

protein

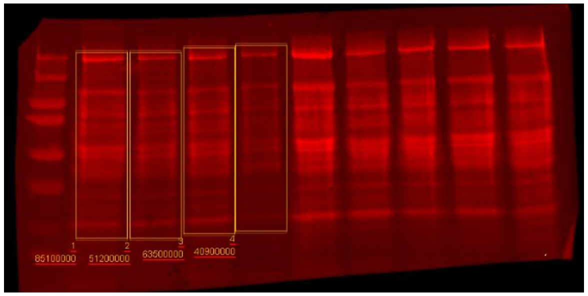

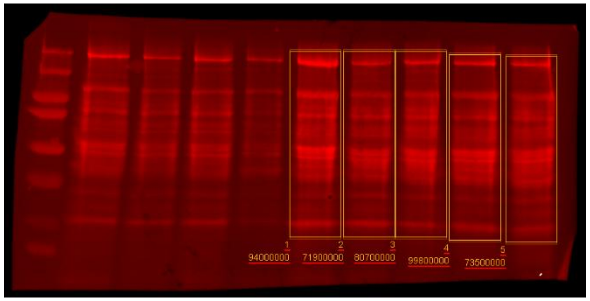

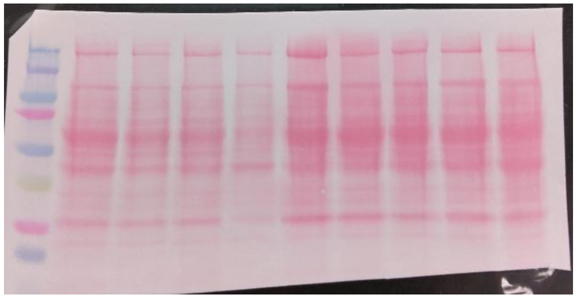

Blot #2 (Lanes: ladder, pooled mesenteric artery, HM3, HM4 HF3, HF4, CM3, CM4, CF3, CF4)

eNOS

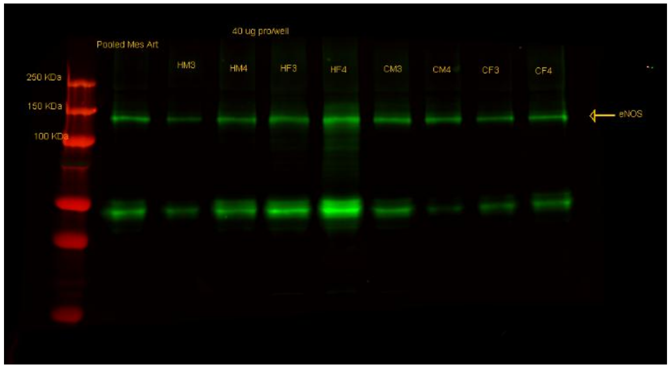

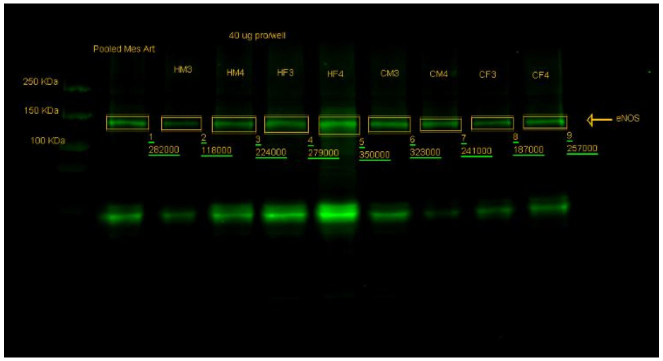

gapdh

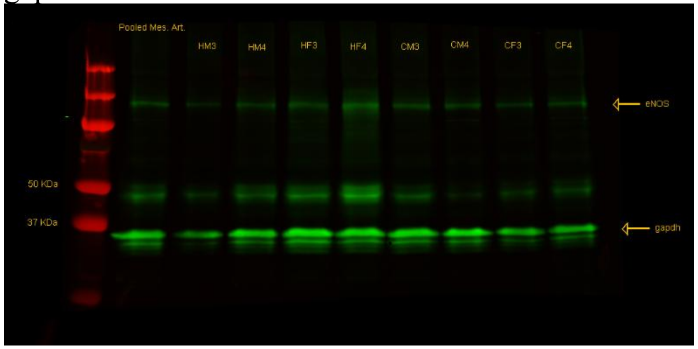

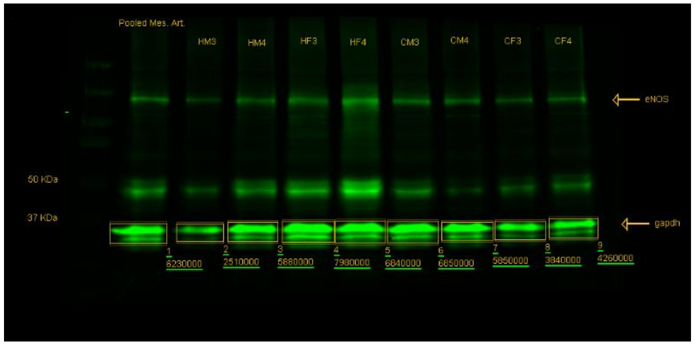

protein

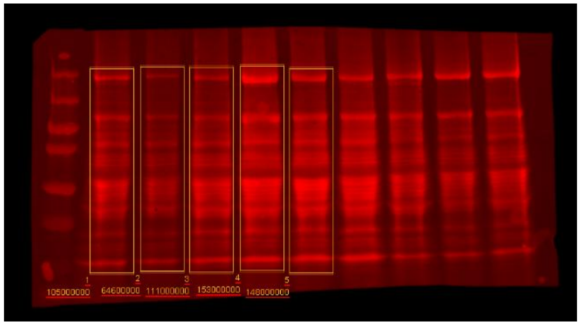

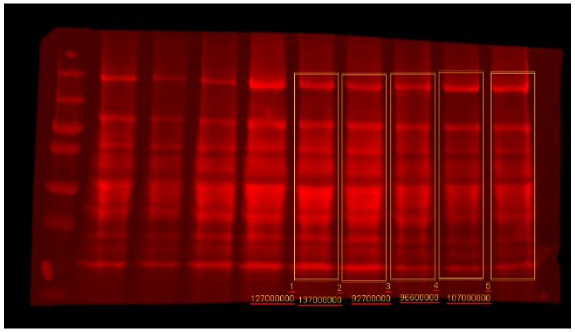

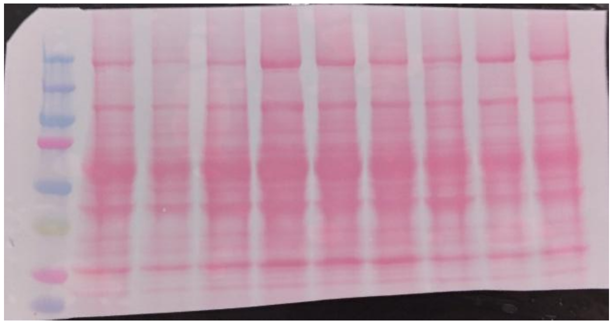

Blot #3 (Lanes: ladder, pooled mesenteric artery, HM1, HF1 CM1, CF1, HM2, HF2, CM2, CF2)

eNOS

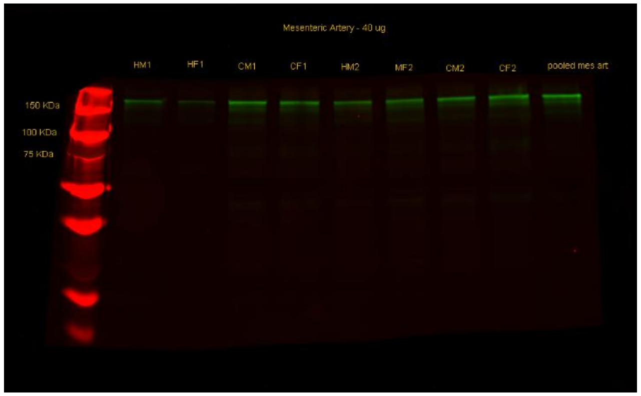

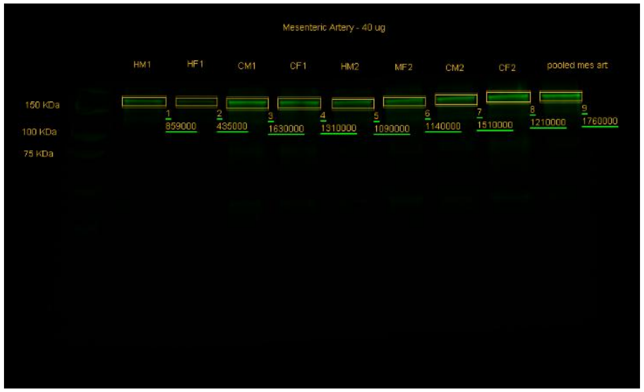

gapdh

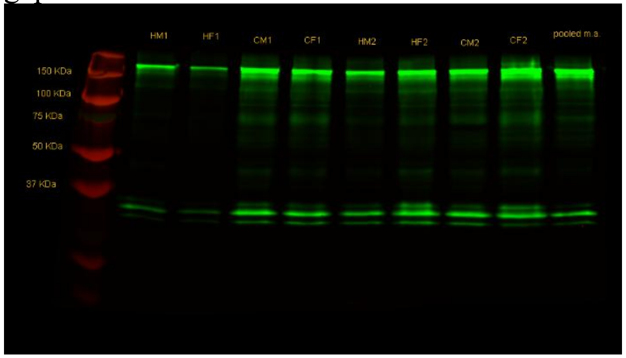

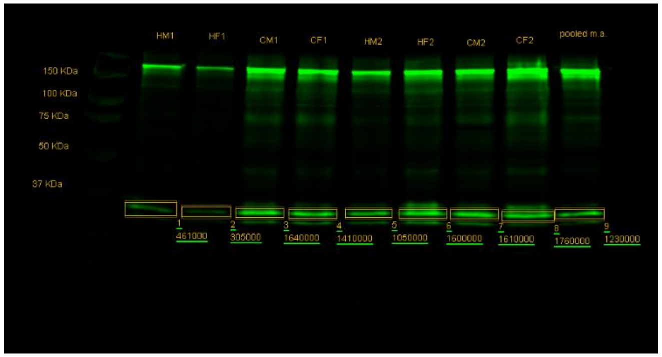

protein

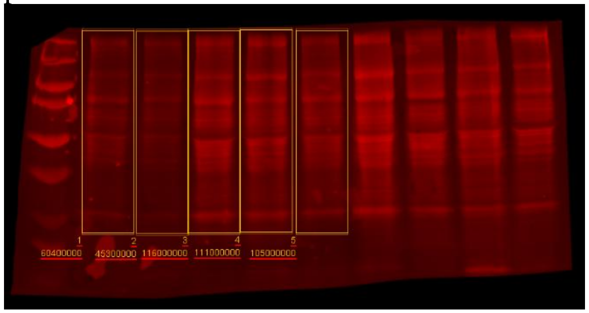
1
2
3
4
5
60400000
45300000
116000000
111000000
105000000

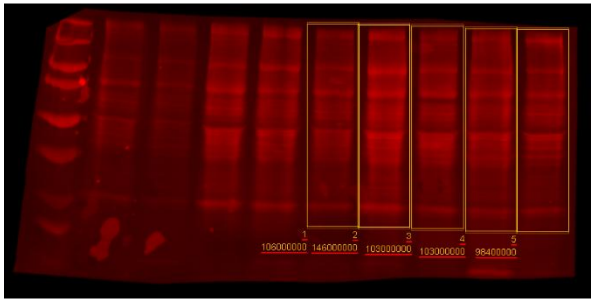
1
2
3
4
5
106000000
146000000
103000000
103000000
98400000

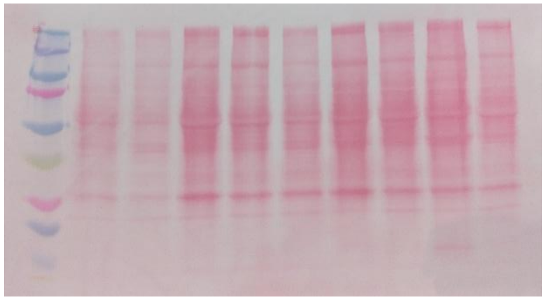

Supplementary Figure 5. Comparison of smallest (<200 µm luminal diameter) and largest (≥250 µm luminal diameter) C57-Male arteries indicate no differences in myoendothelial feedback responses to 10^{-5} M phenylephrine or subsequent vasodilation to 10^{-4} M acetylcholine. Group responses were compared by 2-way repeated-measures ANOVA with Bon Ferroni post-hoc test.

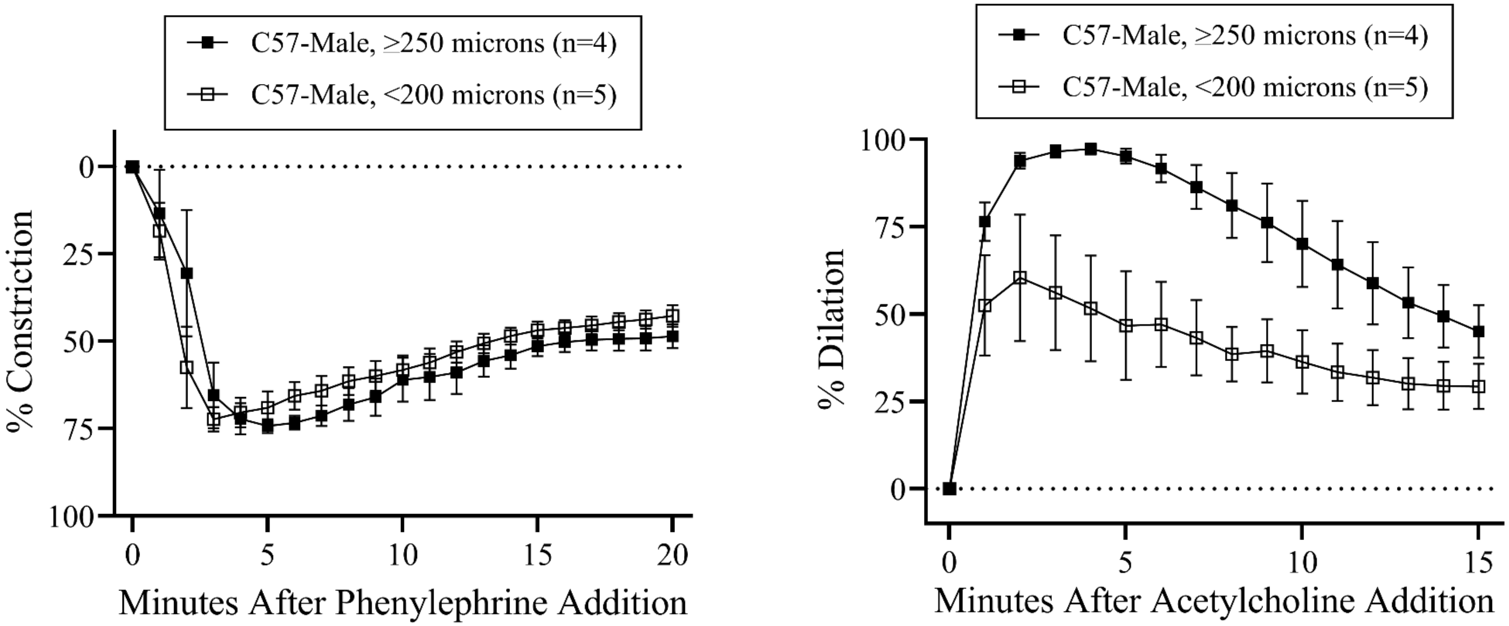

Supplementary Figure 6. Comparison of myoendothelial feedback responses to 10^{-5} M phenylephrine and subsequent vasodilation to 10^{-4} M acetylcholine of mesenteric arteries from youngest (60-78 days old) and oldest (120 days old) HET3-Female mice indicate no significant differences. Group responses were compared by 2-way repeated-measures ANOVA with Bon Ferroni post-hoc test.

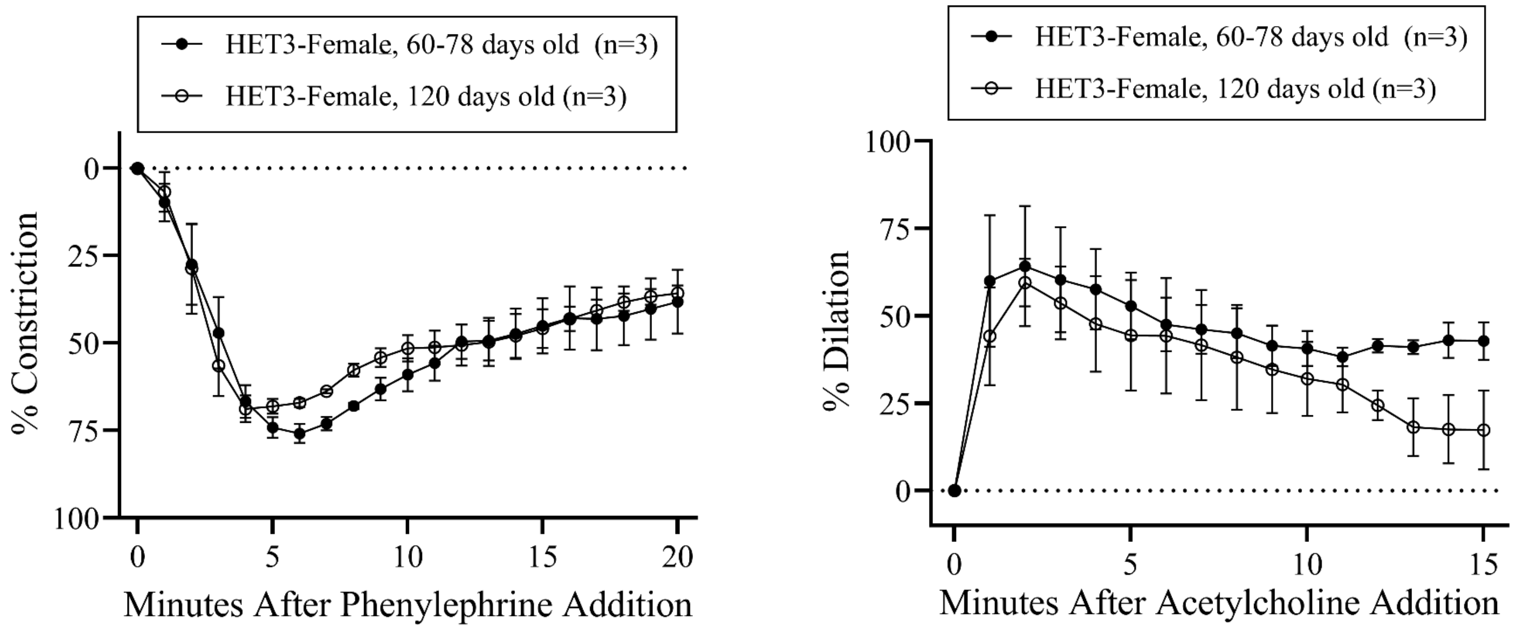

Supplement: Supplementary file 1 [file DataSheet1.pdf]
